# Formation of a super-dense hydrogen monolayer on mesoporous silica

**DOI:** 10.1038/s41557-022-01019-7

**Published:** 2022-08-29

**Authors:** Rafael Balderas-Xicohténcatl, Hung-Hsuan Lin, Christian Lurz, Luke Daemen, Yongqiang Cheng, Katie Cychosz Struckhoff, Remy Guillet-Nicolas, Gisela Schütz, Thomas Heine, Anibal J. Ramirez-Cuesta, Matthias Thommes, Michael Hirscher

**Affiliations:** 1 Max Planck Institute for Intelligent Systems, Stuttgart, Germany; 2 Neutron Scattering Division, Neutron Sciences Directorate, Oak Ridge National Laboratory, Oak Ridge, TN USA; 3 Helmholtz Center Dresden-Rossendorf, Institute of Resource Ecology, Leipzig Branch, Leipzig, Germany; 4 Anton Paar Quantatec Boynton Beach, Boynton Beach, FL USA; 5 Laboratoire Catalyse et Spectrochemiem, Normandie University, ENSICAEN, CNRS, Caen, France; 6 School of Mathematics and Science, TU Dresden, Dresden, Germany; 7 Department of Chemistry, Yonsei University, Seodaemun-gu, Seoul, Republic of Korea; 8 Institute of Separation Science and Technology, Department of Chemical and Biological Engineering (CBI), Friedrich-Alexander University, Erlangen, Germany

**Keywords:** Physical chemistry, Surface chemistry

## Abstract

Adsorption on various adsorbents of hydrogen and helium at temperatures close to their boiling points shows, in some cases, unusually high monolayer capacities. The microscopic nature of these adsorbate phases at low temperatures has, however, remained challenging to characterize. Here, using high-resolution cryo-adsorption studies together with characterization by inelastic neutron scattering vibration spectroscopy, we show that, near its boiling point (~20 K), H_2_ adsorbed on a well-ordered mesoporous silica forms a two-dimensional monolayer with a density more than twice that of bulk-solid H_2_, rather than a bilayer. Theoretical studies, based on thorough first-principles calculations, rationalize the formation of such a super-dense phase. The strong compression of the hydrogen surface layer is due to the excess of surface–hydrogen attraction over intermolecular hydrogen repulsion. Use of this super-dense hydrogen monolayer on an adsorbent might be a feasible option for the storage of hydrogen near its boiling point, compared with adsorption at 77 K.

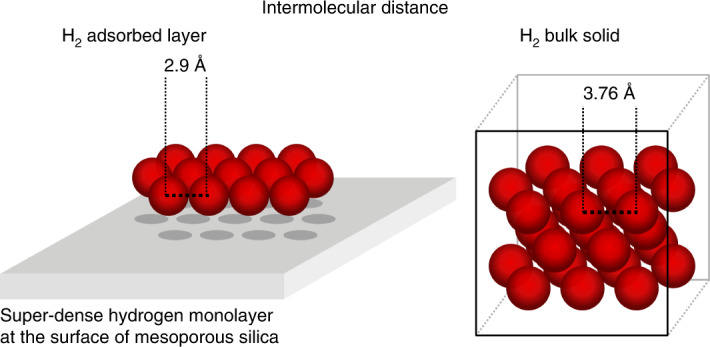

## Abstract (web summary)

In some cases, hydrogen adsorption close to its boiling temperature shows unusually high monolayer capacities, but the microscopic nature of this adsorbate phase is not well understood. Now, H_2_ adsorbed on a well-ordered mesoporous silica surface has been shown to form a 2D monolayer with very short H_2_···H_2_ intermolecular distances and a density more than twice that of bulk-solid H_2._

## Main

In 1949, Schaeffer et al.^1^ reported isotherms with an abnormally high He capacity for the first adsorbate layer on carbon black (Spheron 6 and C) at 4 K. Shortly afterwards, Singh et al.^2^ and Meyer^3^ made similar observations. In 1956, Steele^4^ proposed a phenomenological bilayer model to rationalize the observed high He capacity. In 1959, Pace and Siebert^5^ reported a large discrepancy in the determined surface area of graphon, graphitized carbon black, using nitrogen (77 K) and hydrogen (20 K) adsorption experiments. They suggested an intermolecular distance between hydrogen molecules of 2.95 Å, which implies a density in the adsorbed two-dimensional (2D) layer that substantially exceeds the 3D density of solid hydrogen.

Further efforts to understand the origin of this ‘anomalously high’ monolayer capacity^2^ were made by Huber et al.^6–8^ using a combination of infrared spectroscopy and adsorption measurements of H_2_ on Vycor glass (Supplementary Section 1). Direct comparison of their H_2_ and N_2_ adsorption results implies an intermolecular distance of 3.08 Å, far below that of the bulk solid (3.76 Å), and corresponding to roughly three times the 3D density of liquid hydrogen.

Since the 1950s, several He and H_2_ adsorption experiments have confirmed this anomalously high monolayer capacity on regular surfaces, close to their condensation temperatures (a summary of representative reported values is provided in Supplementary Table 1). Under these conditions, the monolayer capacities for H_2_, D_2_ and He are larger than the capacities of N_2_ or Ar by a factor of around two. Our theoretical–experimental work provides new support for the hypothesis of the existence of a high-density H_2_ layer.

To assess the possible formation of a high-density H_2_ layer, we used, as adsorbent, the ordered mesoporous silica KIT-6. This material consists of a well-defined 3D network of solely mesopores (with a mode pore diameter of 10 nm)^9–12^. The pore diameter is thus 35 times higher than the kinetic diameter of H_2_ (2.89 Å). The surface curvature is large enough to model a flat surface while providing a large surface area. In the absence of micropores, as in KIT-6, pore condensation happens at a higher relative pressure than that required to form the first monolayer. Hence, using argon atoms and hydrogen molecules as a probe at their boiling point, the Brunauer–Emmett–Teller (BET) method can be applied in a straightforward way to accurately determine the monolayer capacity for benchmarking (see the 2015 International Union of Pure and Applied Chemistry (IUPAC) recommendations^13^).

Using high-resolution cryo-adsorption experiments, we confirm the anomalously high adsorption of H_2_ and D_2_ at their boiling temperatures (~20 K) compared with the corresponding Ar (87 K) uptake. The high-flux neutron spallation source at Oak Ridge National Laboratory (ORNL) and the high-resolution vibrational spectrometer VISION, which features an inelastic count rate that is more than two orders of magnitude beyond similar available instruments, allow us to study the formation of the dihydrogen layer in situ by inelastic neutron scattering (INS), with unprecedented resolution. INS has a unique sensitivity to molecular hydrogen, particularly the rotational transitions. First-principles-based model calculations, combined with a simple 2D model representing nuclear quantum effects and path-integral molecular dynamics simulations using density-functional theory rationalize the high-density phase by the relatively small intermolecular repulsion of the compressed hydrogen compared to the surface–adsorbate attraction.

## Results

### High-resolution cryo-adsorption experiments

**Fig. 1:**
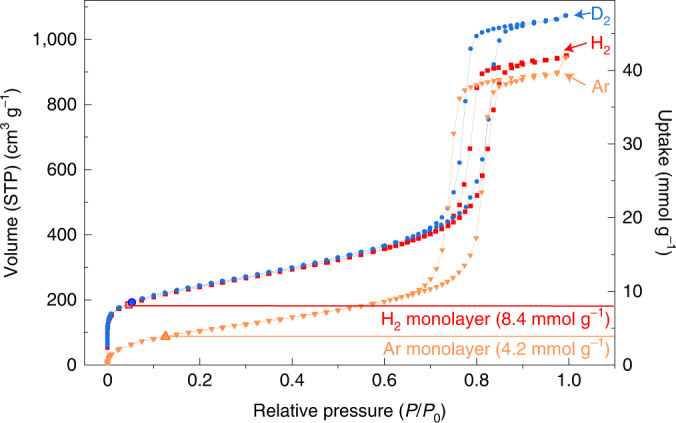
Condensation-temperature adsorption isotherms of H_2_, D_2_ and Ar in KIT-6. H_2_, D_2_ and Ar isotherms measured at their condensation temperatures (20.37, 23.31 and 87.3 K, respectively; left-hand axis) and monolayer capacities according to the BET model for H_2_ and Ar (right-hand axis). All adsorbates show type IV isotherms (Ar, orange triangles; H_2_, red squares; D_2_, blue circles). The H_2_ isotherm shows a monolayer capacity two times higher than that of Ar (8.4 versus 4.2 mmol g^−1^), indicating that two times more H_2_ molecules are necessary to cover the surface compared to Ar atoms. Source data

Figure 1 presents adsorption isotherms of three different gas probes (H_2_, D_2_ and Ar) on KIT-6, measured by high-resolution cryo-adsorption experiments at their condensation temperatures. All isotherms are type IV in the IUPAC isotherm classification^13^, indicative of multilayer adsorption and pore condensation. The subtype H1 hysteresis loop present in the data is typical for capillary condensation in non-constricted cylindrical-like pore channels^14,15^.

Table 1 summarizes the surface characteristics of KIT-6 obtained from the Ar 87 K data. By applying state-of-the-art methods (described in Supplementary Section 2.2 and refs. ^9,16^), it is possible to obtain reliable pore-size and pore-volume information (Supplementary Fig. 2). The pore-size distribution confirms the absence of microporosity for this KIT-6 silica sample. The pore condensation and hysteresis behaviour of H_2_ and D_2_ agree qualitatively with the well-known behaviour of Ar at its boiling temperature. However, both H_2_ and D_2_ show anomalous behaviour in the lower-pressure region of the adsorption isotherm, where monolayer formation occurs. The completion of a statistical monolayer using the BET model allows for an analysis of the monolayer capacity, *n*_m_. A monolayer is the number of molecules necessary to cover the surface completely with a single layer. For H_2_, it yields a value of 8.4 mmol g^−1^, which is about double the measured value for Ar (4.2 mmol g^−1^; Table 1). These values suggest that, close to their boiling temperatures, H_2_ and D_2_ form monolayers on silica with a substantially higher density than the argon adsorbate monolayer at its boiling temperature of 87 K.

**Table 1:** Gas and condensation temperature, monolayer capacity, specific surface area, cross-sectional area and monolayer density in KIT-6

Gas	Temperature (K)	Monolayer capacity (mol g^−1^)	Specific surface area (SSA-BET) (m^2^ g^−1^)	Cross-sectional area (Å^2^)	Intermolecular distance H_2_···H_2_ (Å)	Monolayer density (kg m^−3^)
Ar	87.3	0.0042	359	14.2	–	–
H_2_	20.37	0.0084	719^a^	7.1^b^	2.9	202^c^
D_2_	23.31	0.0086	664^a^	7.0^b^	2.8	413^c^

^a^The specific surface area (SSA) was calculated by using the cross-sectional area 14.2 Å^2^ and 12.9 Å^2^ calculated using hexagonal-close-packing distribution and the bulk-liquid density of H_2_ and D_2_, respectively.

^b^The cross-sectional area was calculated by comparing the monolayer capacity for H_2_ and D_2_ and the surface area determined by Ar (87 K) adsorption experiments.

^c^As a comparison, the corresponding 3D density was calculated by assuming that the H_2_···H_2_ intermolecular distance of the 2D layer extends in all three dimensions, forming a hexagonal close-packing lattice.

### Inelastic neutron scattering

We systematically performed in situ INS experiments to further explore this high-density H_2_ adsorbate layer formation. Stepwise, KIT-6 was exposed to different amounts of *para*-hydrogen (*p*-H_2_) and measured at several temperatures (5, 20.3, 25 and 35 K). For all cases, the dosing steps correspond to a fraction of the BET monolayer coverage (1 ML = 8.4 mmol g^−1^) and can be correlated to a point in the adsorption isotherm (Supplementary Fig. 5).

**Fig. 2:**
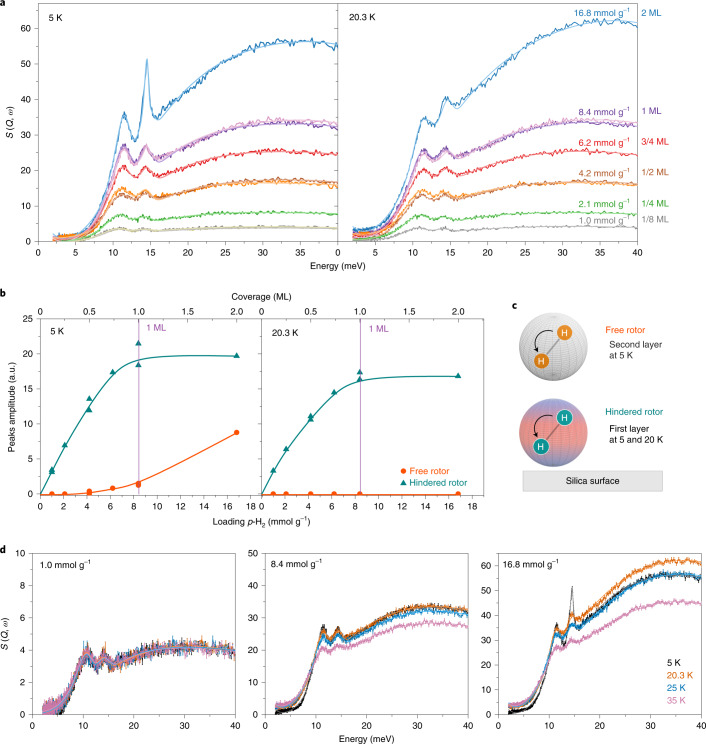
Temperature-dependent INS of different H_2_ loadings. **a**, INS spectra corresponding to a coverage of 1/8, 1/4, 1/2, 3/4, 1 and 2 monolayers at 5 K (left) and 20.3 K (right). All spectra show two low-energy peaks centred at energies close to 11 and 14 meV that correspond to hindered transitions of the free-rotor energy (14.6 meV). The skewed Gaussian analysis shows that the peaks at 11 and 14 meV are the superposition of three transitions centred at 10.6, 11.6 and 14.2 meV. S(Q,ω) is called the ‘scattering law’ and is directly related to the inelastic neutron signal; Q is the momentum transfer and ω is the neutron energy loss. **b**, Peak amplitude as a function of *p*-H_2_ loading obtained from the skewed Gaussian analysis at 5 K (left) and 20.3 K (right) for free rotors (red circles) and hindered rotors (green triangles). The hindered-rotor amplitude saturates near 8.2 mmol g^−1^, indicating the completion of one monolayer. **c**, Representation of the local rotational barrier of the H_2_ molecule in the second layer (isotropic potential) and next to the silica surface (anisotropic potential, represented in pink and blue). In the first layer, the rotational barrier alters the free-rotor transition, splitting it into three transitions (hindered rotor). **d**, Temperature influence on the INS spectra for three different *p*-H_2_ loadings. For loadings of ≤1 ML, the spectra show little temperature dependence, indicating that the H_2_ molecules in the monolayer remain in a similar state up to 25 K. Source data

Figure 2a presents a comparison between the KIT-6 vibrational spectra collected at 5 and 20.3 K after dosing different coverages of *p*-H_2_ (1/8, 1/4, 1/2, 3/4, 1 and 2 ML), as well as the respective curve fittings. At both temperatures, the spectra have two low-energy peaks centred at energies close to 11 and 14 meV, with a very broad maximum at ~30 meV corresponding to molecular recoil of the hydrogen molecule^17,18^. The spectra at 1/8, 1/2 and 1 ML were collected in two separate experiments, and show excellent reproducibility of the INS spectra shape. We used a skewed Gaussian peak for each transition (Supplementary Figs. 6–8 provide details). The analysis determines that the peaks at 11 and 14 meV are the superposition of three transitions centred at 10.6, 11.6 and 14.2 meV. A narrow peak at 14.6 meV is present at 5 K, corresponding to a contribution from the free-rotor transition of the H_2_ molecule to the spectra (Supplementary Fig. 9).

Figure 2b shows the amplitude (area) of the peak-fitting analysis as a function of loading. The area of the peaks centred at 10.6, 11.6 and 14.2 meV (green) grows with increasing loading up to 1 ML at all temperatures, reaching a defined saturation at 1 ML. The fourth, narrow, peak (orange), centred at 14.6 meV, is apparent above 1 ML. Figure 2d compares the spectra at 1/8, 1 and 2 ML for all temperatures. Interestingly, the spectra intensity, which usually decreases with temperature, has little dependence on the temperature at the lowest *p*-H_2_ loading and starts to decrease with temperature for higher *p*-H_2_ dosings. This indicates that the H_2_ molecules up to 1 ML remain in a similar state up to 25 K.

To interpret the transitions observed in the INS spectrum of adsorbed hydrogen, we used a 3D rigid-rotor model subjected to a hindering potential (rotational barrier). The rotational barrier splits the free-rotor transition of H_2_ (Supplementary Fig. 10) into several transitions depending on the symmetry of the rotational barrier. The rotational levels can be obtained by solving the Schrödinger equation using the spherical harmonics as the basis set^19^ (details are provided in Supplementary Section 5.2). The calculated transition energies as a function of rotational barrier heights are shown in Supplementary Fig. 11. The rotational barriers *V*_ϕ_ = 0.4 meV (ϕ, azimutal angle) and *V*_θ_ = 7.7 meV (θ, polar angle) yield three transitions at 10.6, 11.6 and 14.2 meV, which agree with the experimentally obtained energies (Supplementary Figs. 8 and 9).

### Path-integral molecular dynamics simulation

**Fig. 3:**
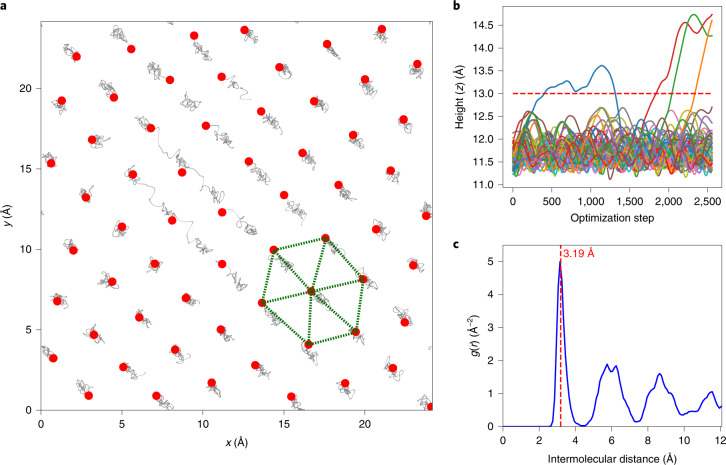
PIMD simulation for H_2_ adsorbed on a flat silica surface. **a**, PIMD path of motion (grey): the initial configurations of the H_2_ molecules on a silica surface correspond to an MD equilibrated configuration. The final positions (red dots) of the H_2_ molecules show a hexagonal pattern. **b**, The height (*z* axis) of all H_2_ molecules as a function of the optimization step (each colour represents a molecule), showing three H_2_ molecules leaving the surface. **c**, Pair distribution function (g(r)) for H_2_ molecules on the surface: the first neighbours’ distance (intermolecular distance) is close to 3.2 Å, which is reasonably close to the experimental findings (2.9 Å). Source data

To further support our experimental findings, we performed ab initio simulations. In the case of Ar on SiO_2_, we used molecular dynamics (MD), whereas for H_2_ on SIO_2_ we performed path-integral molecular dynamics (PIMD)^20^, where nuclear quantum effects were considered for the light-weight adsorbed H_2_ molecules (for more details, see Methods and Supplementary Section 7). Our simulation assumes a simple flat silica surface, Si_72_O_144_ (24 × 24 Å), with molecules/atoms adsorbed on top of the surface (details are given in Supplementary Section 7). The Ar MD calculations equilibrate to an adsorbed layer on top of silica composed of 42 atoms (Si_72_O_144_ + Ar_42_). The Ar atoms form a hexagonal arrangement with an intermolecular distance of 3.91 Å (Supplementary Fig. 18), close to that of bulk-liquid Ar (4.07 Å). In the case of H_2_, PIMD calculations show the same silica surface covered by an adsorbed layer containing 63 H_2_ molecules (Si_72_O_144_ + 63H_2_), with an average intermolecular distance of 3.2 Å (Fig. 3), far below the bulk-liquid (4.05 Å) and bulk-solid (3.76 Å) dihydrogen intermolecular distances. For both simulations, the hexagonal arrangement of the adsorbed atoms/molecules results in an incommensurate phase (Supplementary Figs. 18 and 19); that is, the arrangement does not correspond with the disposition of the silica atoms. The total number of adsorbed atoms or molecules can be compared directly, because both simulations were made using the same silica surface. The coverage ratio H_2_/Ar is 1.6, which is lower than our experimental value obtained by adsorption (Supplementary Table 1).

### Theoretical analysis

**Fig. 4:**
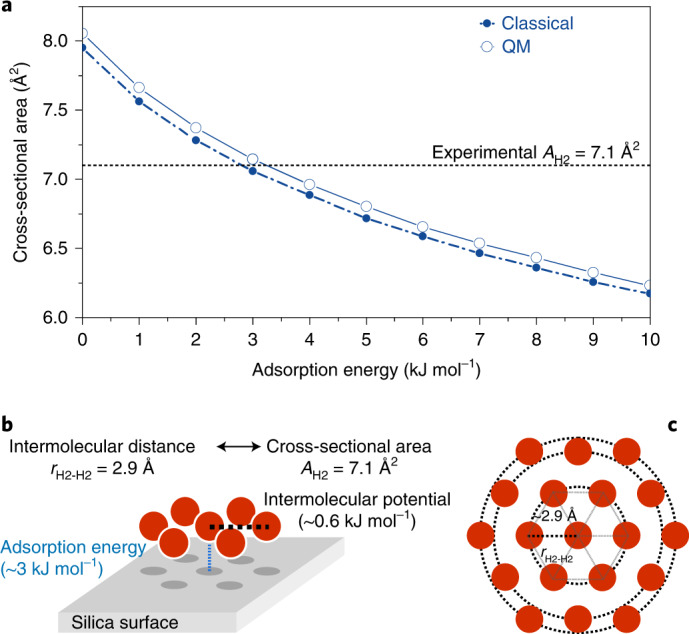
Ab initio calculation of the hydrogen cross-sectional area as a function of adsorption energy. **a**, Minimum cross-sectional area per molecule depending on the strength of the adsorption potential. The dashed lines and circles represent the simulation in a classical picture, and the solid lines and circles show the solution including quantum effects (QM). For an adsorption energy of ~3 to 4 kJ mol^−1^, the calculated cross-sectional area *A* is close to the experimental value of 7.1 Å (equivalent to a H_2_···H_2_ separation of 2.9 Å). **b**, Scheme of the average intermolecular potential (0.6 kJ mol^−1^) for an averaged silica adsorption energy of 3 kJ mol^−1^. **c**, Scheme of the intermolecular separation for a silica adsorption energy of 3 kJ mol^−1^. Source data

We consider a molecule adsorbed on a flat surface surrounded by other molecules of the same kind (2D model) to estimate the cross-sectional area per H_2_ molecule. The Hamiltonian can be written as

$${H} = - \frac{{{\hbar}^2}}{2m}{\Delta} + {\underbrace {{\sum} {{{ V}_{\rm{M}}}{\left( {{\bf{r}} - {{\bf{r}}_{i}}} \right)} + {{ V}_{\rm{s}}}} }_{: = {V}_{\rm{tot}}{\left( {\bf{r}} \right)}}}$$

where ${{V}}_{\rm{M}}\left( {\bf{r}} \right)$ represents the intermolecular interaction, here modelled by the spherical symmetric Morse potential, **r**_*i*_ denotes the positions of the surrounding molecular centres, and *V*_s_ is the adsorption potential of the surface (details are provided in Supplementary Section 6.1). A Morse potential is fitted by ab initio results obtained at the CCSD(T)/aug-cc-pVQZ level^21^. We calculated the cross-sectional area against the adsorption energy by combining these two potential contributions by solving the Schrödinger equation, treating dihydrogen as a featureless particle (Fig. 4a). The stronger the adsorption energy, the more condense the packing becomes. For the experimentally observed area of 7.1 Å^2^ per adsorbed dihydrogen (equivalent to a H_2_···H_2_ separation of 2.9 Å), an adsorption energy of ~3 to 4 kJ mol^−1^ is required.

## Discussion

We have presented a systematic investigation of the phase behaviour of H_2_ and D_2_, close to their boiling temperatures, when adsorbed on ordered mesoporous silica (KIT-6). The observed pore condensation and hysteresis behaviours of H_2_ and D_2_ in the higher relative pressure range of the adsorption isotherms are consistent with the adsorption and phase behaviour of Ar. However, deviations are observed in the region of monolayer formation where, contrary to Ar, H_2_ and D_2_ form super-dense adsorbate phases.

Gas adsorption isotherms at the boiling point are commonly treated using BET theory, which, in a first step, allows determination of the (statistical) monolayer capacity. In a second step, the specific surface area can be calculated by a reasonable assumption of the cross-sectional area, which describes the characteristic area that an adsorbate atom or a molecule occupies on the surface. Typically, the statistical monolayer is assumed to comprise a hexagonal-close-packed 2D layer with bulk-liquid density^22^. For Ar, the bulk-liquid density yields a cross-sectional area of 14.2 Å^2^, and analysis of the isotherm results in a specific surface area of 359 m^2^ g^−1^ for KIT-6 (Supplementary Fig. 3). However, assuming bulk-liquid density, H_2_ and D_2_ yield much higher surface areas, almost twice that of the Ar measured area (Supplementary Section 2.3). On the other hand, in the mesoporous structure of KIT-6 without any microporosity, the accessible surface area is the same for all used gases. Therefore, given that both gases see the same surface, the difference in the monolayer capacities implies the existence of a super-dense hydrogen monolayer.

From the determined monolayer capacity of H_2_ and the surface area determined by Ar, we found a molecular cross-sectional area for adsorbed H_2_ of 7.1 Å^2^ (Supplementary Section 2.4). Assuming that the H_2_ molecules form a 2D layer with a hexagonal distribution, the corresponding H_2_···H_2_ intermolecular distance on the surface is 2.9 Å. This is a surprisingly low value. If this 2D intermolecular distance extended in all three dimensions, forming a hexagonal close-packing lattice, the 3D density would be 202 kg m^−3^. This density exceeds the bulk-liquid and bulk-solid densities of hydrogen (*ρ*_lq_ = 70.9 kg m^−3^ and *ρ*_solid_ = 80.0 kg m^−3^) by a factor of almost three. Similarly, the D_2_ adsorption isotherm analysis yields exceptionally high monolayer density with a cross-sectional area, *A*_D2_, of 7.0 Å^2^ (Table 1).

Two straightforward explanations for these high monolayer capacities for hydrogen isotopologues are possible. Either dihydrogen adsorbs as bilayers rather than monolayers (the Steele model^4^) or the H_2_ molecules become strongly compressed when adsorbed on the surface.

Further proof of the super-dense state of the dihydrogen adsorbate is obtained by comparison of the INS spectra with our model calculations. The H_2_ molecules on the first layer are seen as hindered rotors^18,23^ by the INS. Accordingly, the hindered-peak amplitude saturation indicates that the formation and completion of the first layer of adsorbed molecules occur between 0 and 8.4 mmol g^−1^. This saturation value corresponds precisely with the monolayer capacity measured independently by gas adsorption. The second and further layers are no longer influenced by the surface and appear as free rotors^18,24^ in the 5-K data, and are not seen at 20.3 K and higher temperatures (Fig. 2b). Because the samples were loaded at the corresponding points on the adsorption isotherms, it is clear that there is no experimental evidence for the existence of a bilayer. It follows that all the hydrogen at monolayer capacity is in direct contact with the surface, demonstrating the presence of a super-high-density layer of adsorbed hydrogen.

Another observation of the INS experiments is that the adsorbed phase does not change between 5 and 20 K for *p*-H_2_ loadings below the monolayer capacity (8.4 mmol g^−1^), that is, below and above the freezing point of bulk hydrogen (Fig. 2d), suggesting an immobile state of the adsorbate within this range of loading.

Our computational analysis shows that the strongly compressed layer agrees with first-principles calculations. The intermolecular interactions of the dihydrogen-KIT-6 system are given solely by intermolecular van-der-Waals interactions (Fig. 4b, intermolecular dihydrogen–dihydrogen and KIT-6 surface–dihydrogen), and the intermolecular Pauli repulsion takes place when two molecules or a molecule and the surface get too close to one another. Given the small polarizability of dihydrogen compared to other gases or the KIT-6 surface atoms, the attractive interactions are governed by the surface–dihydrogen interactions. These are on the order of 3–4 kJ mol^−1^ and thus strongly exceed the intermolecular dihydrogen potential of 0.6 kJ mol^−1^. Due to the small energy scale and the absence of core electrons, H_2_ molecules can be compressed easily. This is reflected in the high compressibility of bulk liquid and solid dihydrogen^25^. PIMD was used to calculate the structural properties of a simplified version of this quantum system at a finite temperature (Methods and Supplementary Section 7). Our simulation results show that an incommensurate hexagonal layer is adsorbed independently on the surface atom arrangement, with an intermolecular distance H_2_···H_2_ of 3.2 Å, higher than the experimental value found (2.9 Å). However, it shows that a flat layer of silica can reproduce the extremely high densities of a H_2_ adsorbed layer. The comparison between Ar and H_2_ simulations shows a coverage ratio of 1.6, indicating the substantial difference in the number of molecules occupying the same surface.

Furthermore, we refine the intermolecular distance by treating the H_2_ adsorbed molecules as featureless quantum particles (Methods and Supplementary Section 6). Our numerical model reflects that a compressed 2D dihydrogen layer forms when the KIT-6–dihydrogen adsorption energy is 3–4 kJ mol^−1^, a typical value for silica surfaces. These results agree with the assumption of a single-layer adsorbate of dihydrogen.

## Conclusion

We have presented a comprehensive study of the layer formation of H_2_ adsorbed close to its boiling temperature (20.3 K) on ordered mesoporous silica (KIT-6). Our adsorption data analysis shows that the surface is occupied by about twice the number of H_2_ (D_2_) molecules compared to Ar. Although the observed pore condensation and hysteresis behaviour for H_2_ and D_2_ resemble Ar at 87 K, there are substantial deviations between H_2_ (D_2_) and Ar adsorption in the lower-pressure range where monolayer formation occurs. In other words, we find that the density of this monolayer substantially exceeds the density of bulk-liquid H_2_ (D_2_). This can be derived from a determination of the monolayer capacity by the BET method, which can be applied straightforwardly.

This study confirms early reports on the high-density adsorption of light-weight molecules and atoms such as H_2_ and He on surfaces, leading to about twice the surface coverage compared to their heavier counterparts, such as Ar. The exceptionally high layer density can be explained based on our thorough experimental–theoretical investigation results and does not require assumptions such as instantaneous adsorption of double layers, as made by Steele^4^.

The super-high density is a consequence of the high compressibility of the first-row gases H_2_ and D_2_ (and He) due to the absence of core electrons in these elements. As the adsorption energy at the surface substantially exceeds the intermolecular interactions, the surface layer is compressed to a value that is about twice the amount expected for uncompressed adsorbates (bulk-liquid density). Spectroscopic experimental confirmation of the super-high-density H_2_ and D_2_ layers was achieved with INS vibration spectroscopy. The observed hindered-rotor (5 and 20 K) and free-rotor (5 K) intensities clearly mark the completion of a monolayer of H_2_ on the silica surface by quantitatively determining the number of molecules covering the surface, hence proving that hydrogen does not form a bilayer, but instead forms a super-high-density monolayer.

The super-high-density layer hypothesis is in line with the observed adsorption behaviour, with the three low-energy peaks observed in the INS experiments and with the results of the calculations. The high compressibility of the H_2_ adsorbate layers on surfaces needs to be considered for quantitative analysis of H_2_ adsorption isotherms at 20 K.

Hydrogen storage and transport play a key role in an energy economy based on renewables. Liquid-phase hydrogen storage is now receiving serious consideration for various applications because of its high volumetric density. Adsorbent-based hydrogen storage in porous high-surface-area materials has mainly been investigated at 77 K, but this results in a relatively low volumetric density. Formation of this high-density 2D hydrogen may potentially increase the volumetric capacity of cryogenic hydrogen-storage systems. Our low-pressure hydrogen adsorption isotherms for temperatures between 20 and 77 K (Supplementary Fig. 1c) show a dramatic increase in hydrogen uptake by lowering the temperature; at 600 mbar between 77 and 20 K, this increase is equivalent to one order of magnitude (Supplementary Fig. 1c,d). Therefore, lowering the operating temperature of a cryo-adsorbent tank could yield a substantial increase in volumetric storage density, making it competitive with liquid-phase hydrogen storage. Our results are based on a model system, KIT-6, a well-characterized ordered mesoporous silica with smooth connected surfaces. The newest high-porosity materials, metal–organic frameworks (MOFs) with BET areas of 5,000 m^2^ g^−1^ and more, which show the highest gravimetric hydrogen uptake at 77 K, possess rather fragmented inner surfaces with different chemical compositions. Hydrogen adsorption isotherms of MOFs at 20 K are scarcely available in the literature. However, two examples^26,27^ show a lower difference between hydrogen and nitrogen adsorption at their respective boiling points. This may indicate that the super-dense hydrogen monolayer cannot form on highly fragmented surfaces. Further studies will be needed to elucidate the exact structural properties required for this super-high-density hydrogen-layer formation.

## Methods

### Low-pressure high-resolution cryo-adsorption experiments

A fully automated Sieverts apparatus was used to perform the adsorption experiments. The calibration cell was an empty analysis carried out at the same temperature and pressure range as the experiment. The calibration curve was automatically subtracted from the data, and minor corrections related to the sample volume and the nonlinearity of the adsorbate were made. A coupled cryocooler based on the Gifford–McMahon cycle was used to control the sample temperature. The cooling system allowed us to measure temperatures from 20 to 300 K with an estimated error of <0.05 K. The pore size distribution was calculated from the isotherm experiments for argon using non-local density-functional theory (Supplementary Section 2).

### Inelastic neutron scattering

Inelastic neutron scattering experiments were performed at the vibrational spectroscopy beamline VISION at SNS, ORNL (Spallation Neutron Source, Oak Ridge National Laboratory). A cryocooler cycle system was used to control the temperature of the sample in a range of 5–100 K. The *para*-hydrogen was prepared by liquefying ultrahigh-purity normal hydrogen over Oxisorb (CrO nanoparticles deposited over silica) at 17 K and bleeding the vapour of this system at 22 K. All samples were dried in vacuum, overnight at 150 °C. The background signal associated with the sample holder and the sample itself was subtracted (Supplementary Fig. 4). Different loadings of *para*-hydrogen (1/8, 1/4, 1/2, 3/4, 1 and 2 ML), corresponding to a fraction of the monolayer coverage (1 ML = 0.84 mmol g^−1^), were used to follow the monolayer formation process.

### Details of first-principles calculations

The intermolecular potential was calculated assuming a spherical symmetric Morse potential. The Morse potential was fitted with ab initio results at the CCSD(T)/aug-cc-pVQZ level. The relative orientation of the molecules towards each other was also taken into account. The cross-sectional area was calculated under the following assumptions: (1) each H_2_ molecule binds with the same adsorption energy to the surface (0–10 kJ mol^−1^); (2) the H_2_ molecules are frozen (that is, the intramolecular bond length is fixed); (3) the total potential felt by an adsorbed H_2_ molecule is given by the superposition of the fitted Morse potentials of surrounding H_2_ molecules plus the adsorption potential of the silicate surface; (4) quantum effects are included and the zero-point energy levels are calculated for each considered energy of adsorption (Supplementary Section 6).

### Details of the simulations

MD and PIMD simulations were performed using CP2K (http://www.cp2k.org)^28^, based on the mixed Gaussian and plane-wave scheme^29^ and the Quickstep module^30^. The calculations used the molecularly optimized double-zeta-valence plus polarization (DZVP) basis set^31^, Goedecker–Teter–Hutter pseudopotentials^32^ and the Perdew–Burke–Ernzerhof (PBE) exchange correlation functional^33^. The plane-wave energy cutoff was 400 Ry. The DFT-D3-level correction for dispersion interactions, as implemented by Grimme et al.^34^, was applied, with a cutoff distance of 15 Å. For hydrogen molecules on silica, the temperature for the simulation was 30 K, and the time step was 1 fs. DFT PIMD simulations were performed using ring polymer molecular dynamics (RPMD) with a path integral Langevin equation (PILE) thermostat and 32 beads per atom^35^. The MD equilibrated configuration was used as the starting point, and 2,500 steps were simulated. The adsorbate surface was assumed to be a flat silica surface (Si_72_O_144_; Supplementary Section 7).

## Online content

Any methods, additional references, Nature Research reporting summaries, source data, extended data, supplementary information, acknowledgements, peer review information; details of author contributions and competing interests; and statements of data and code availability are available at 10.1038/s41557-022-01019-7.

## Supplementary information

Supplementary Information Supplementary Table 1, Figs. 1–19 and Discussion.

## Data Availability

Supplementary Information is available for this paper. The source data for the figures and Supplementary Information can be found at the Zenodo repository (10.5281/zenodo.6623388)^36^. The data that support the findings of this study are available online and from the corresponding authors upon reasonable request. Correspondence and requests for materials should be addressed to R.B.X. and M.H. Source data are provided with this Paper.

## Source data

Source Data Fig. 1 Ar, H_2_ and D_2_ adsorption isotherms data.
Source Data Fig. 2 Compressed folder with ‘.csv’ files of all INS results including spectra and analysis and source data of Fig. 2b..
Source Data Fig. 3a PIMD initial and final positions.
Source Data Fig. 3b z-position of H_2_ molecules for all optimization steps.
Source Data Fig. 3c Pair distribution function data.
Source Data Fig. 4a H_2_ cross-sectional area as a function of adsorption potential.
