# Supplementary material for: Formation of a super-dense hydrogen monolayer on mesoporous silica
Source: Nat Chem. 2022 Aug 29;14(11):1319–24. doi: 10.1038/s41557-022-01019-7 (PMC9630099; doi:10.1038/s41557-022-01019-7)
Supplement: Supplementary file 1 — Supplementary Table 1, Figs. 1–19 and Discussion. [file 41557_2022_1019_MOESM1_ESM.pdf]

nature portfolio

https://doi.org/10.1038/s41557-022-01019-7

Supplementary information

Formation of a super-dense hydrogen monolayer on mesoporous silica

In the format provided by the authors and unedited

Supplementary Information

Formation of super-dense hydrogen monolayer on mesoporous silica

Rafael Balderas-Xicohténcatl[1,2], Hung-Hsuan Lin[3], Christian Lurz[3], Luke Daemen[2], Yongqiang Cheng[2], Katie Cychosz Struckhoff[4], Remy Guillet-Nicolas[5] , Gisela Schütz[1], Thomas Heine[3,6,7], Anibal J. Ramirez-Cuesta[2], Matthias Thommes[8], Michael Hirscher[1]

[1]Max-Planck Institute for Intelligent Systems, Heisenbergstrasse 3, 70569 Stuttgart, Germany
[2]Neutron Scattering Division, Neutron Sciences Directorate, Oak Ridge National Laboratory, Oak Ridge, Tennessee 37831, United States
[3]Helmholtz Center Dresden-Rossendorf, Institute of Resource Ecology, Leipzig Branch, Permoserstr. 15, 04318 Leipzig, Germany
[4]Anton Paar Quantatec Boynton Beach, FL 33426, United States
[5]Normandie University, ENSICAEN, CNRS, Laboratoire Catalyse et Spectrochemiem 14050 Caen, France
[6]Chair of Theoretical Chemistry, School of Mathematics and Science, TU Dresden, 01062 Dresden, Germany.
[7]Department of Chemistry, Yonsei University, Seodaemun-gu, Seoul 120-749, Republic of Korea
[8]Institute of Separation Science and Technology, Department of Chemical and Biological Engineering (CBI) Friedrich-Alexander University, Erlangen 91058, Germany

Contents

1. Literature results on H_2 and He adsorption at low temperature
2. High-resolution adsorption experiments
   2.1. Adsorption analysis data
   2.2. Pore volume and pore size
   2.3. BET analysis
   2.4. Layer density and cross-sectional area
3. Inelastic neutron scattering (INS) experiments
   3.1. INS dosing steps
   3.2. INS temperature dependence
4. INS gaussian analysis
5. Para-hydrogen inelastic neutron spectra
   5.1. Solid and liquid hydrogen, free rotor
   5.2. Roto-vibrational transitions, hindered rotor
6. Theoretical analysis
   6.1. Simulation of cross-sectional area
   6.2. Fitting Morse potential parameters
7. MD and Path Integral Molecular Dynamics simulations
   7.1. Argon adsorption simulation
   7.2. Hydrogen adsorption simulation
8. Data availability
9. Supplementary references

1. Literature results on H_2 and He adsorption at low temperature

Table S1. Reported values of the monolayer capacity of light gases (He and H_2) adsorbed at low temperatures compared to N_2 or Ar monolayer values.

	Material	Monolayer capacity (N_2 77K)	Specific surface area (N_2 77K)* $m^2\ g^{-1}$	Monolayer capacity (H_2, He)	Specific surface area * $m^2\ g^{-1}$	Coverage ratio † ($n_m(H_2)$ / $n_m(N_2)$)
Schaeffer *et al.* *(1949).*[1]	Carbon black (Spheron 6)	129.5 cc/g	113	He (4.2K) 310.5 cc/g	He (4.2K) 258	2.2
Schaeffer *et al.* *(1949).*[1]	Carbon black (Spheron C)	260.5 cc/g	227.8	He (4.2K) 569.5 cc/g	He (4.2K) 472.7	2.1
Pace and Siebert(1959)[2]	Graphon	0.9 mmol/g**	85.9	H_2 (20.4K) 2.0 mmol/g**<br>D_2 (23.5K) 2.2 mmol/g**	H_2 (20.4K) 169.3<br>D_2 (23.5K) 174.3	2.2
Brewer and Champeney *(1962)*[3]	Vycor glass disk	25.9 cc/g	113	He (4.22K) 62.5 cc/g<br>He (3.15K) 66.8 cc/g<br>He (2.02K) 75.3 cc/g<br>He (1.79K) 78.3 cc/g	--	2.4<br>2.6<br>2.9<br>3.0
Huber and Huber(1987)[4,5]	Vycor glass	2.1 mmol/cc	--	H_2 (18K) 3.5 mmol/cc	--	1.7
Setoyama and Kaneko(1995)[6]	Zeolite NaY	126 molecules/unit cell	--	He (4.2K) 223 molecules/unit cell	--	1.8
Setoyama and Kaneko(1995)[6]	Zeolite KL	10 molecules/unit cell	--	He (4.2K) 21.8 molecules/unit cell	--	2.2
Edler et al.(1997)[7]	MCM-41	10.0 mmol/g	980	H_2 (21.5K) 15.8 mmol/g	--	1.6
Tanaka *et al*(2004).[8]	SWCH	9.6 mmol/g	938	H_2 (20K) 15.7 mmol/g	1353	1.6
Prisk *et al.*(2014)[9]	MCM-41	10.0 mmol/g	979	H_2 (19.4K) 15.7 mmol/g	--	1.6
**This work**	KIT-6	Ar (87K) 4.2 mmol/g	Ar (87K) 359	H_2 (20.3K) 8.4 mmol/g<br>D_2 (23.3K) 8.6 mmol/g	H_2 (20.3K) 719<br>D_2 (23.3K) 664	H_2(20.3K)/Ar(87K) 2.0<br>D_2(23.3K)/Ar(87K) 2.0

* Surface areas estimated by use of the cross-sectional areas, which were calculated from the respective bulk liquid density and hexagonal close-packing N_2 (16.2 Å^2), Ar (14.2 Å^2), H_2 (14.2 Å^2), D_2 (12.9 Å^2) and He (15.4 Å^2).
**Calculated based on the reported surface areas by Pace and Siebert[2].
† Calculated for this work under the assumption that both molecules see the same surface area, the monolayer capacity ratio indicates how many more molecules (He or H_2) cover the surface compared to Ar or N_2.

Several reports of He and H_2 adsorption experiments at temperatures close to the condensation temperature have shown an "anomalously high" monolayer capacity for regular surfaces such as carbon, silica, or zeolites. Table S1 shows a comparison of monolayer capacities measured by N_2 or Ar with the monolayer capacities measured by a light gas H_2, D_2, or He close to boiling temperatures, respectively. The difference in monolayer capacity yields a large difference in the reported surface areas measured by different gases, calculated in each case assuming a layer density close to bulk liquid.

The ratio between monolayer capacities of the light gases (H_2, D_2, or He) and their heavier counterparts (N_2 or Ar) was calculated for all reported values, and it represents how large

is the coverage of H_2 (D_2 or He) compared to N_2 or Ar. In all cases, the monolayer capacity of the light gas is at least 1.5 times larger compared to those of N_2 or Ar.

Assuming that the light gases see the same surface area that N_2 or Ar, the monolayer capacity ratio indicates how many more molecules of H_2 (D_2 or He) cover the same surface compared to Ar or N_2. Our results using the mesoporous silica KIT-6 are included for comparison, yielding a ratio of H_2/Ar close to 2. Interestingly, Brewer and Champeney *(1962)*[3] obtained a helium monolayer at 1.7 K that corresponds to a ratio (He/N_2) as high as 3.

Huber *et al.*[4,5,10] studied this difference between H_2 and N_2 adsorption using a combination of infrared spectroscopy and adsorption measurements of H_2 on Vycor glass. The ratio (H_2/N_2) that can be obtained from their adsorption results yields 1.7. Furthermore, they interpreted the coverage dependence of intramolecular H_2 transition as the first evidence of a bilayer formation[4,5,10]. However, Vycor glass and the choice of N_2 as a probe molecule (for obtaining reference/benchmark data) are less than an optimal choice for studying the possible formation of a high-density hydrogen adsorbate surface phase. Owing to nitrogen's quadrupole moment and resulting specific interactions with polar surface functionality (such as hydroxyl groups, which are present on the Vycor glass surfaces), the orientation of the adsorbed N_2 molecule on the surface, and consequently its cross-sectional area, is not well defined (it can vary between 16.2 and 13.5 $Å^2$ as shown in ref.[11]). Besides, Vycor glass consists of a highly disordered pore system, exhibiting larger micropores and narrow mesopores[12,13] which does not allow for a straightforward and reliable application of the Brunauer–Emmett–Teller (BET) method for surface area determination (see 2015 IUPAC recommendations[14]). Furthermore, it introduces a high level of complexity into the infrared spectra, complicating its interpretation.

2. High-resolution adsorption experiments

A fully automated Sieverts' apparatus (Quantachrome iQ_2) was used to perform the adsorption experiments. The device is equipped with three different pressure transducers ($1\cdot10^{-5}$-0.1, 0.1-10, 10-1000 torr) to accurately measure the pressure in the range $1\cdot10^{-8}$-1

bar. A closed-cycle cryocooler is used to control the sample temperature in the range 19.5-300 K with an estimated error of ±0.05 K. The gas purity is 99.999 % for Ar, N_2, H_2, and 99.8 % for D_2.

2.1 KIT-6 adsorption analysis data

Before the adsorption experiments, 80 mg of KIT-6 sample were activated overnight in high vacuum at 150 °C. Prior to each experiment, the sample was kept in high vacuum overnight at room temperature.

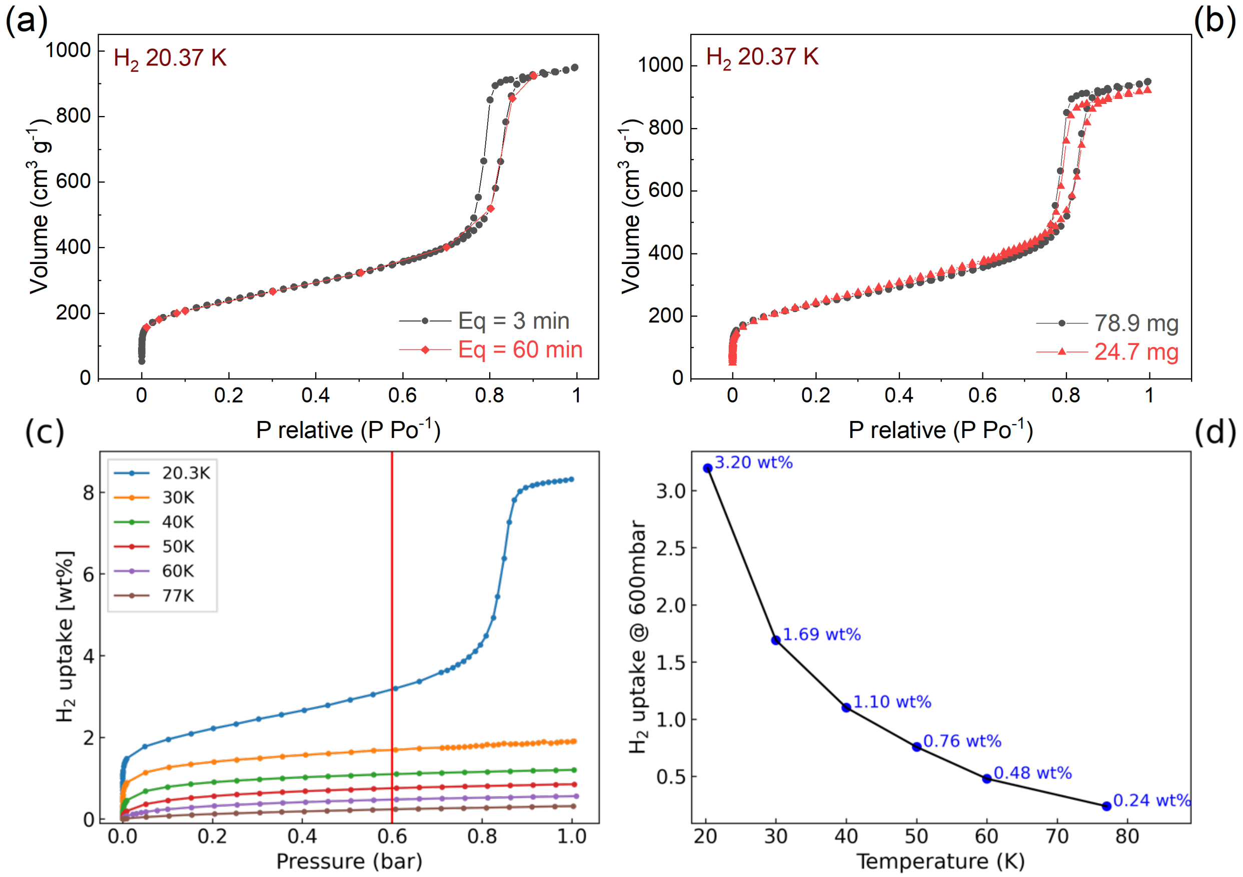

Figure S1. Low-temperature hydrogen adsorption isotherms. (a) At 20.37 K using different equilibrium times of 3 and 60 minutes per point in the isotherm. **(b)** At 20.37 K using different sample masses. In both cases, (a) and (b) a high reproducibility can be observed indicating neither any difference in the total adsorbed amount nor in the low-pressure region where the monolayer is completed. **(c)** Hydrogen adsorption isotherms (uptake wt%.) at different temperatures. **(d)** H_2 uptake at 600 mbar as a function of temperature, indicating that at subcritical temperatures, the H_2 uptake is increased almost one order of magnitude.

Figure S1a shows two KIT-6 hydrogen adsorption isotherms collected at 20.37 K for a different number of points and different equilibration times (3 and 60 min). Both isotherms show an identical uptake, which indicates an adequate thermal and sorption equilibrium at each isotherm point. Figure S1b shows a comparison of the KIT-6 hydrogen adsorption isotherms collected for different masses (24 and 79 mg). There is no difference in the adsorbed amount at low relative pressures ($P/P_0 < 0.1$) where typically the monolayer completion takes place, and only a small variation is observed at high relative pressures ($P/P_0 > 0.9$).

Figure S1c shows the H_2 isotherms of KIT-6 for different temperatures between 20 and 77 K. The uptake values taken at a pressure of 600 mbar (Fig.S1d) are strongly increased by lowering the temperature. The pressure is chosen well below the starting of pore condensation at ~800 mbar and therefore, this enormous increase in the H_2 capacity can be ascribed to the formation of a high-density adsorbed layer at temperatures close to the boiling point of H_2. At higher temperatures, e.g, 77 K, the uptake drops by one order of magnitude compared to 20 K.

2.2 Pore volume and pore size distribution

The KIT-6 pore volume for each isotherm was calculated using the Gurvich's rule[15] close to condensation pressure ($P/P_0 = 0.975$) to be: Ar (1.137 $cm^3\ g^{-1}$), H_2 (1.189 $cm^3\ g^{-1}$), D_2 (1.161 $cm^3\ g^{-1}$). In this case, the four gases occupy a similar volume when the pores are completely filled.

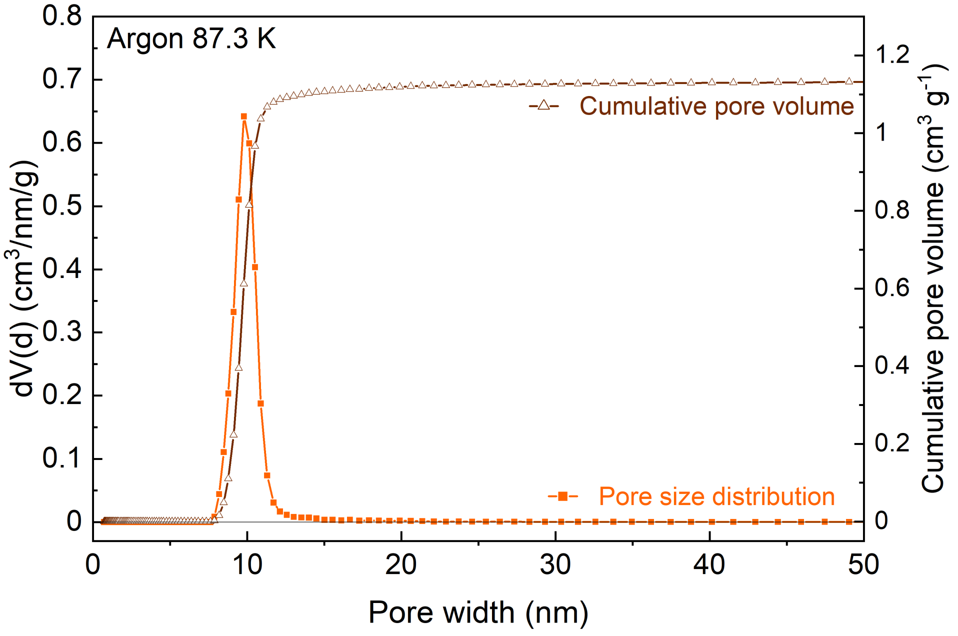

Figure S2. Pore size distribution obtained using NLDFT analysis. Pore size distribution calculated using NLDFT and Ar adsorption isotherms at the condensation temperature 87.3 K. There is only a well-defined peak centered at 10 nm and no contribution from lower sizes, indicating the absence of microporosity.

The pore size distribution (SI Fig. S2) was calculated using the adsorption isotherms and non-local density functional theory (NLDFT) [DFT here refers to the theory of the thermodynamics of inhomogeneous fluids built around the grand free energy and Helmholtz free energy expressed as functionals of the density distribution in the system, not to be confused with electronic DFT, see refs [16,17].]. Argon (87.3 K) shows a well-defined peak centered at 10 nm and no microporosity. The cumulative pore volume value is 1.14 cm^3 g^{-1}, identical to the one obtained using the Gurvich's rule. NLDFT was applied using the kernels contained in the software ASiWwin v3.0 by Quantachrome (Ar - zeolite/silica 87 K) assuming cylindrical pores.

2.3 BET analysis

The KIT-6 BET monolayer capacity and BET surface area were calculated from the isotherms of the three gases and the results are included in Table S1. Figure S3a shows the BET analysis of the argon isotherm calculated with the relative pressure range (0.05 - 0.3), yielding a monolayer capacity of 4.2 mmol g^{-1}, an interaction constant of C = 30, and a specific surface area of 359 m^2g^{-1} determined using a cross-sectional area of Ar, 14.2 Å^2

(ρ_{lq}=1395 kg m^{-3}). The argon cross-sectional area was calculated using the assumption of a bulk-liquid density in the adsorbed phase and a hexagonal-close-packed 2D layer (Eq. 1).

On the other hand, the BET analysis of the H_2 (Fig. S3b) and D_2 isotherms yields a monolayer capacity of 8.4 and 8.6 mmol g^{-1}, respectively. Using the same assumption of a hexagonal-close-packed 2D layer with a bulk liquid density, the H_2 (ρ_{lq}=70.9 kg m^{-3}) and D_2 (ρ_{lq}=163.8 kg m^{-3}) cross-sectional areas are 14.2 and 12.9 Å^2, respectively. Such large discrepancies in the monolayer capacities of H_2 and D_2 (compared to Ar) yield a much higher BET area of 719 m^2 g^{-1} and 664 m^2 g^{-1}, respectively, which is almost twice the Ar BET area.

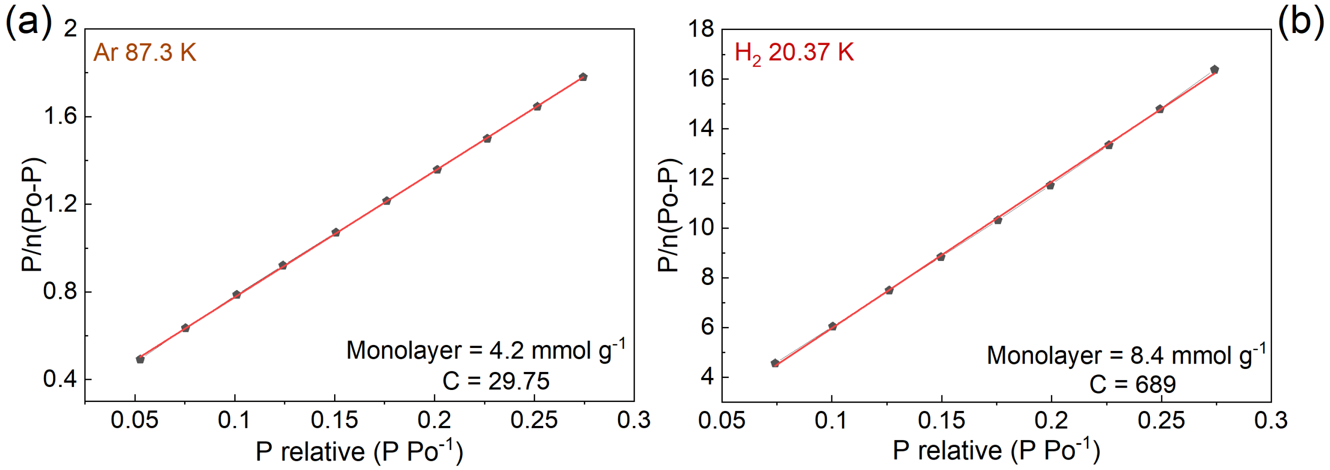

Figure S3. Argon (a) and hydrogen (b) BET plot of KIT-6 at condensation temperature, respectively. The linear fitting was obtained in the relative pressure range (0.05 -0.3) and the parameter C was positive. In both cases, the experiment data increases linearly with relative pressure. The analysis indicates a monolayer capacity of 4.2 and 8.4 mmol g^{-1} for Ar and H_2, respectively.

2.4 Layer density and H_2 cross-sectional area

The hydrogen cross-sectional area, A_{H_2}, can be experimentally obtained by comparing the H_2 monolayer capacity to the specific surface area measured by argon at 87.3 K using the relation

$$S_{Ar} = n_m \cdot A_{H_2} \cdot N_A \qquad \text{(Eq. 1)}$$

where S_{Ar} is the specific surface area determined by Ar and N_A the Avogadro's number.

The H_2 cross-sectional area, A_{H_2}, represents the mean area occupied by a single molecule and is directly correlated to the hydrogen monolayer density by the relation

$$A_{H_2} = f \cdot \left({}^{m}/_{\rho N_A} \right)^{2/3} \qquad \text{(Eq. 2)}$$

with the packing factor, *f*, (*f* =1.091) for a hexagonal-close-packed layer, the molar mass, *m*, and the density of monolayer, ρ [18,19].

The cross-sectional area is also connected to the intermolecular distance, which represents the distance center-center of two adsorbed molecules (H_2-H_2), by the relation:

$$A_{H_2} = \frac{\sqrt{3}}{2} r^2 \qquad \text{(Eq. 3)}$$

where A_{H_2} is the cross-sectional area and r is the intermolecular separation.

For KIT-6, the analysis of the H_2 adsorption isotherm and SSA = 359 m^2g^{-1} yields a cross-sectional area A_{H2} = 7.1 $Å^2$ and a monolayer density of 202 kg m^{-3}, exceeding the bulk liquid density (ρ_{lq}=70.9 kg m^{-3}) and the bulk solid density (ρ_{solid}=80.0 kg m^{-3}) of hydrogen. This cross-sectional area corresponds to an intermolecular separation of 2.9 Å.

3. Inelastic neutron scattering (INS) experiments

Before the INS experiments, 1.213g of KIT-6 sample were activated overnight in high-vacuum at 150°C. Figure S4 shows the INS spectrum of the aluminum sample holder and sample that was subtracted from all experiments at the corresponding temperature.

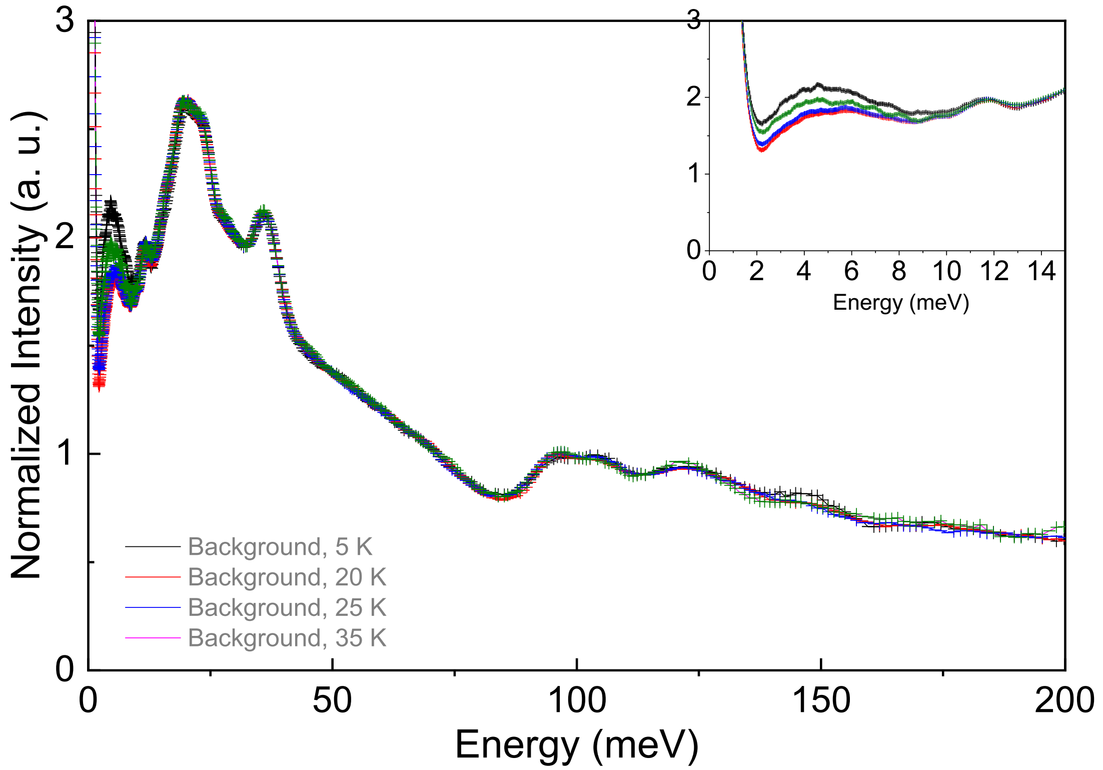

Figure S4. Sample background spectra collected using an aluminum sample holder for the temperatures 5, 20, 25, and 35 K. The 5 K signal shows a larger signal in low energies related to the temperature differences between the cryocooler system and the sample.

3.1 INS dosing steps

Different amounts of hydrogen were dosed corresponding to a fraction (1/8, 1/4, 1/2, 3/4, 1ML) of the monolayer (1ML = 8.4 mmol g^{-1}). For better comparison to the hydrogen adsorption experiments, the dosing points have been marked in the isotherm (Fig. S5)

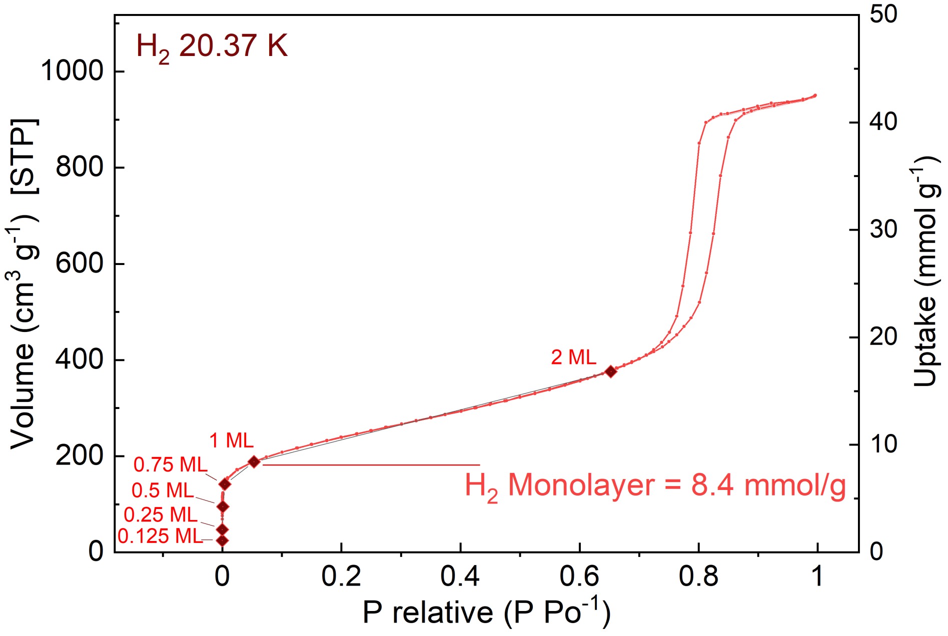

Figure S5. KIT-6 hydrogen adsorption isotherm at 20 K. The monolayer formation process was followed using inelastic neutron scattering. The dosing points per spectra recorded are marked as fractions of the monolayer coverage.

3.2 Temperature dependence

The temperature dependence of the INS spectra has been studied between 5 and 35 K (Fig. 3c). For the lowest loading of 1/8 ML, the spectra of all peaks are temperature independent. However, higher loadings lead to a stronger decrease of the peak's intensity with increasing temperature. At one monolayer (1 ML) coverage, no significant difference in intensities and shape of the spectra collected at 5 and 20 K is observed, while the intensity reduces considerably at 25 and 35 K owing to partial desorption of hydrogen molecules. At 2 ML, the spectrum at 5 K has an overall lower intensity compared to 20 K due to a different recoil of the solid phase of hydrogen compared to liquid, and at 25 and 35 K, the intensity reduces owing to partial desorption of hydrogen molecules.

4. INS gaussian analysis

The INS spectra were analyzed by using a combination of a Skewed Gaussian model for the peaks (10.6, 11.6, and 14.2 meV) and a polynomial of order 6 for the background

(recoil). The asymmetry in the peaks is related to the energy distribution of the neutron beam and is determined by the neutron source and experimental setup. Our analysis considers this asymmetry, and it is included in the parameter γ of the Skewed Gaussian model, where γ = -2.745 for all spectra. For each dosing step, the intensity of the peaks *i.e.* the amplitude of the Gaussian and the energy (center) were obtained. Fig. S6 shows the INS spectra and the respective fitting for all temperatures (5, 20.3, 25, 35 K) and all dosing. In all cases, we observed that the overall intensity increases with increasing dosing, which corresponds linearly with the amount of dosed p-H_2.

Figure S7 show several typical examples of the fitting components for the two primary analyzed temperatures (5 and 20.3 K). The INS spectra show three peaks of the same amplitude centered at 10.6, 11.6, and 14.2 meV. At 5 K, they show an additional narrow peak centered at 14.6 meV. Figures S8 and S9 show some additional parameters of the fitting for 5 and 20.3 K, respectively.

The peaks at 10.6, 11.6, and 14.2 meV are present already at the lowest p-H_2 coverage (1/8 ML) and are characteristic of the first adsorbed layer of hydrogen on the silica KIT-6. The dihydrogen interaction with the polar surface of silica imposes a rotational barrier to the adsorbed molecules located in the first layer which is explained by the hindered-rotor model. Hence, the three signals apparent in the INS spectrum arise from a single layer of hydrogen on the surface, the first adsorbed layer. The amplitude of these three peaks (hindered-rotor transitions) was obtained from the peak analysis and increases linearly with the p-H_2 loading up to the 8.4 mmol g^{-1}, and then it remains constant. This indicates the completion of the first layer of adsorbed molecules that occurs at 8.4 mmol g^{-1}. This saturation value corresponds exactly with the monolayer capacity measured independently by gas adsorption.

A fourth low-energy sharp peak at 14.6 meV is additionally observed for the spectra collected at 5 K (Fig. 3a,b), far below the melting point of hydrogen (14.05 K). This peak corresponds to the free-rotor transition, indicative of the presence of multilayer. At this temperature, the spectroscopic signature of adsorbed dihydrogen in the second layer

resembles the bulk-solid. Hence, the free-rotor peak is associated with the second or higher adsorbed layers and starts to appear only at p-H_2 dosing higher than 8.4 mmol/g, which is consistent with the completion of the first layer observed by the hindered peaks in the INS, and adsorption isotherms. The free rotor transition is not observed at 20.3 K and higher temperatures for dosing higher than 8.2 mmol/g (1ML) (second layer). At temperatures higher than the melting point (20.3, 25, and 35 K), the second adsorbed layer resembles the bulk liquid phase (see Fig. S10).

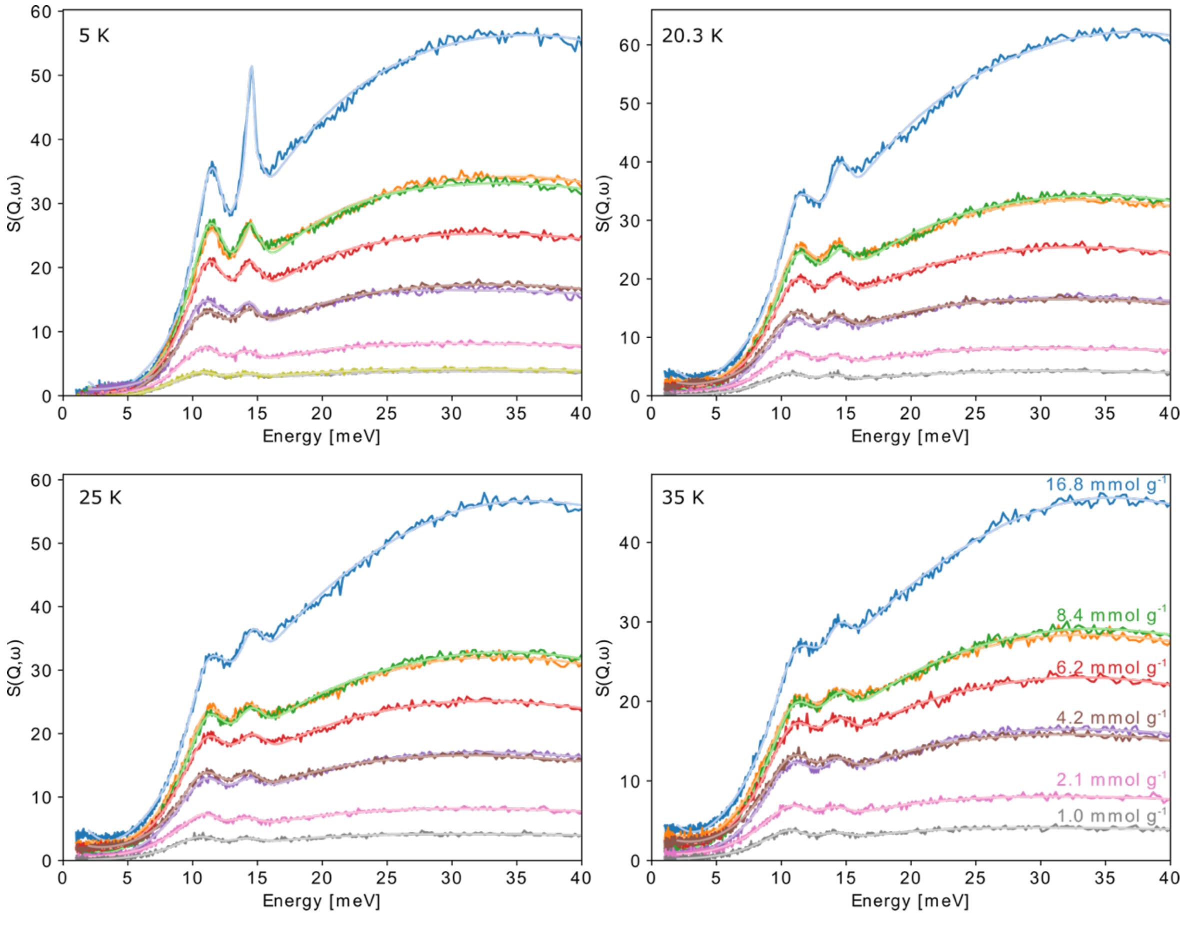

Figure S6. Gaussian fitting for all spectra collected using different p-H_2 dosing at 5, 20.3, 25, and 35 K. The spectra were collected by subsequent dosing steps of p-H_2 corresponding to the surface coverages 1/8, 1/4, 1/2, 3/2, 1 and 2 ML (8.4 mmolg-1). The experiments using 4.2 and 8.4 mmol/g were repeated to corroborate reproducibility. For all temperatures, all spectra show several transitions near 14.6 meV and a broad maximum at 32 meV related to the recoil of the H_2 molecule. Below the melting point (5 K) at the highest dosing (2 ML), the spectra show a sharp peak near 14.6 meV.

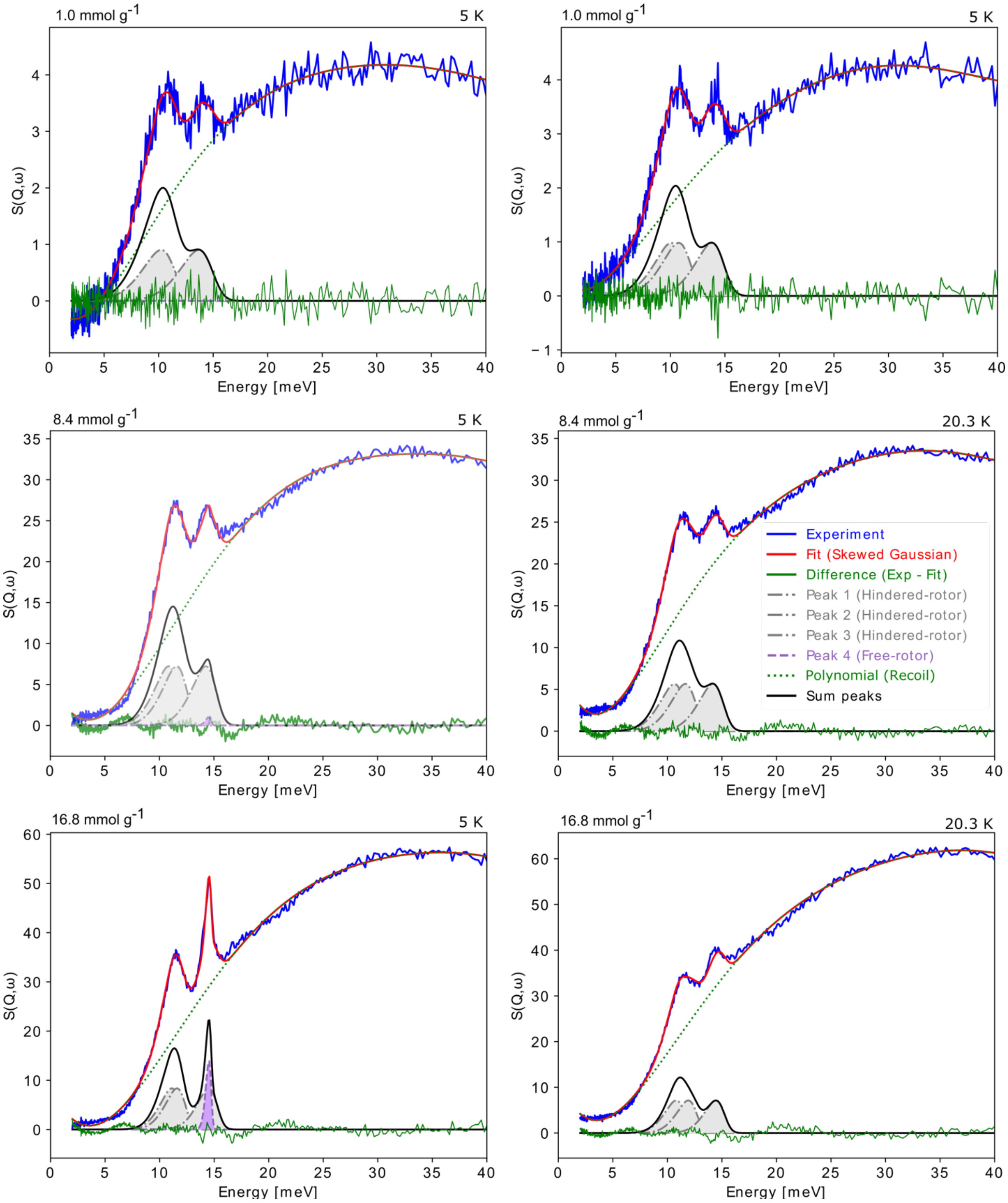

Figure S7. An example of the gaussian analysis of the INS of KIT-6 at 5 K (left) and 20 K (right). The analysis was made assuming that all hindered transitions (gray) have the same probability, and hence same intensity. Our analysis reveals that the two peaks at 11 and 14 meV are composed of the sum of three peaks centered close to 10.6, 11.6, and 14.2 meV. The recoil broad maximum at 32 meV was modeled by a polynomial of order 6 or 7. A four peak at 14.6 meV appears at 5K and high p-H_2 dosing (left-bottom, purple).

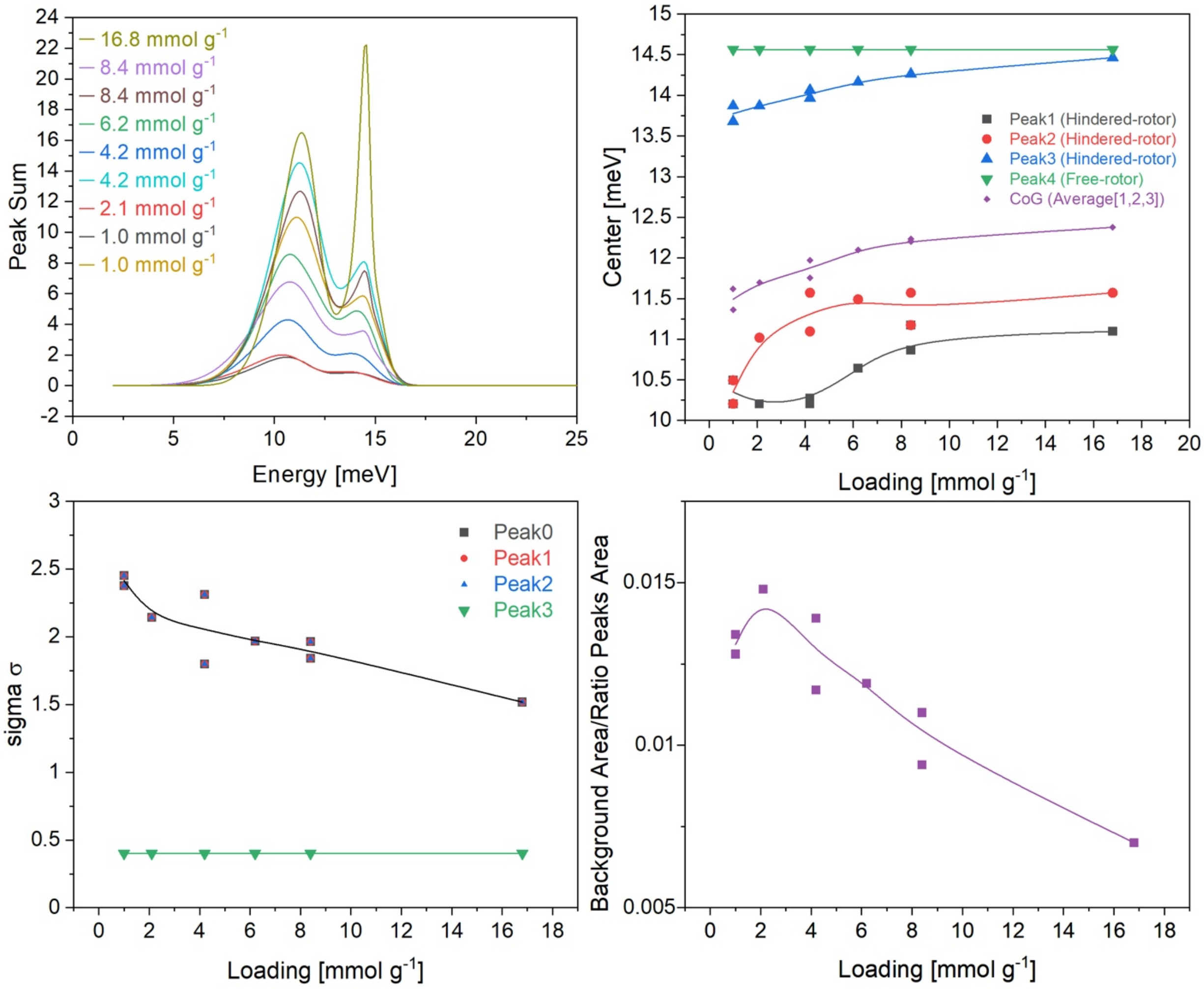

Figure S8. Fitting parameters of the Skewed-Gaussian analysis for different p-H_2 dosing at 5 K. The center of the three peaks is shifted to higher energies with increasing coverage up to 1 ML where the peaks remain centered at 10.6, 11.6, and 14.2 meV, respectively (upper-left).

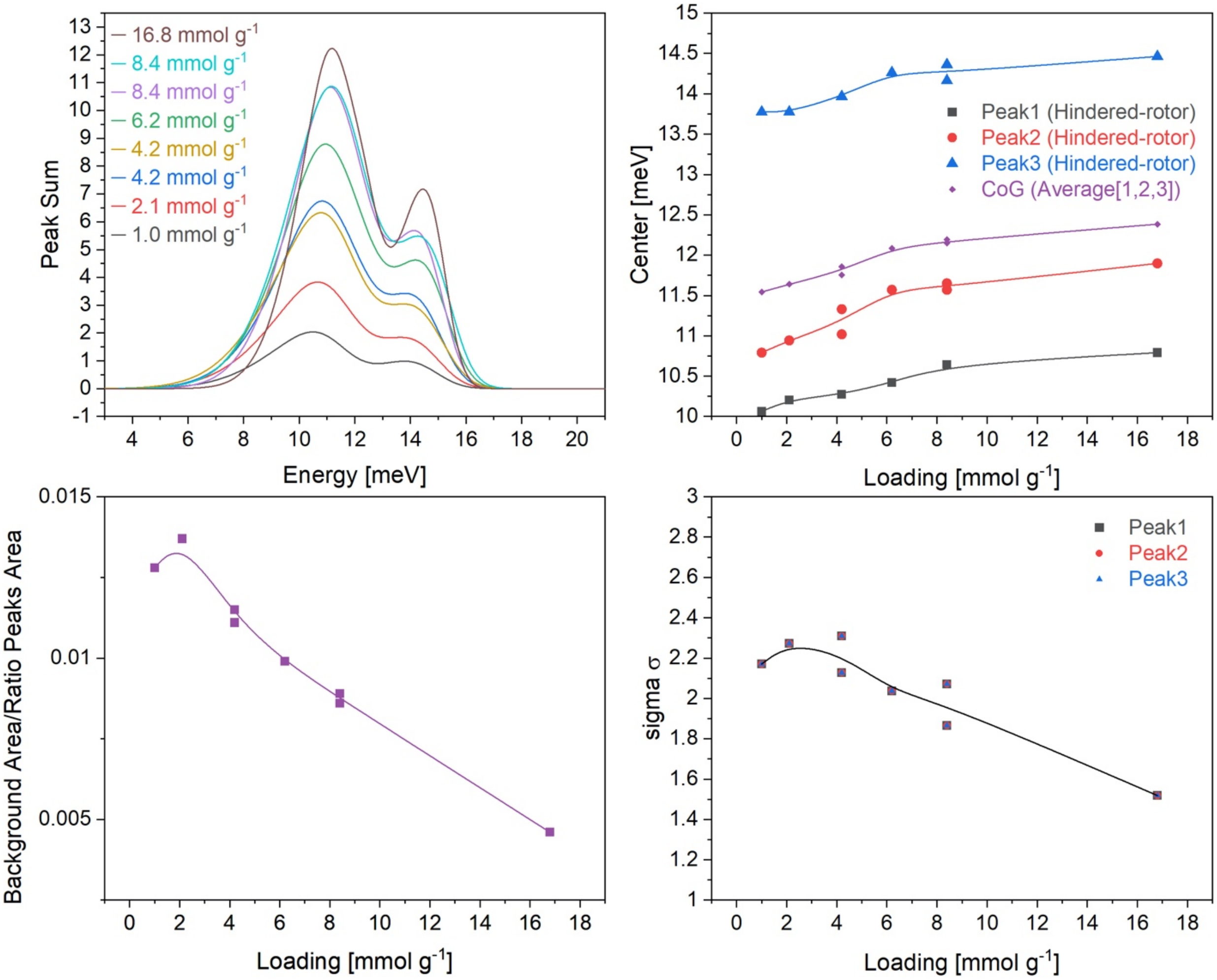

Figure S9. Fitting parameters of the Skewed-Gaussian analysis for different p-H_2 dosing at 20 K. The center of the three peaks is shifted to higher energies with increasing coverage up to 1 ML where the peaks remain centered at 10.6, 11.6, and 14.2 meV, respectively (upper-left).

5. Para-hydrogen inelastic neutron spectra

5.1 Bulk solid and bulk-liquid hydrogen INS spectra

In solid and liquid state, hydrogen possesses a remarkable difference (Fig. S10a); in solid-state, the hydrogen molecules behave as a free rotor with energy states associated with the rotational motion of a complete isolated molecule. Because hydrogen molecules in solid-state are all almost unperturbed by any interaction between the neighboring hydrogen molecules (quantum solid), they can show a rotational transition at 14.6 meV; as solid

hydrogen is warmed the effects of the recoil become more marked. In liquid-state, the molecular interactions H_2-H_2 are larger, the rotational transitions disappear and only the recoil signal (32 meV) remains in the INS[20,21].

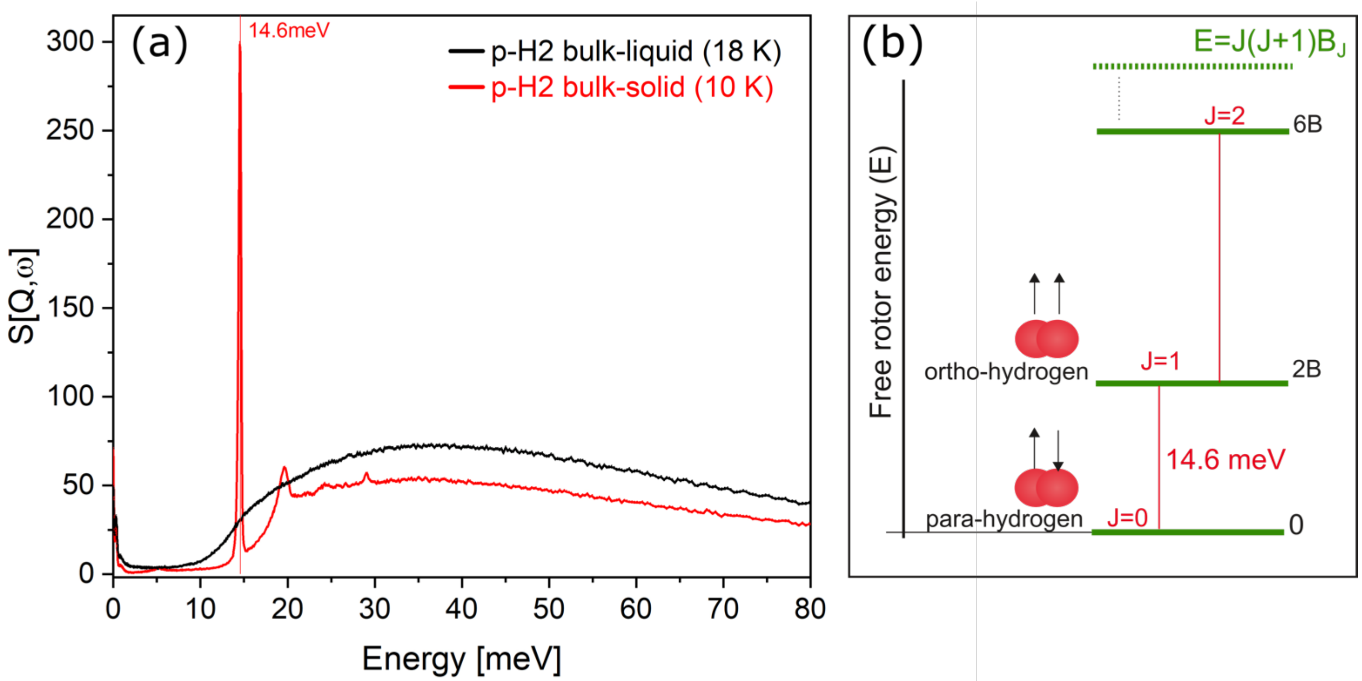

Figure S10. INS spectra of bulk solid and liquid H_2. (a) Vibrational neutron spectra of solid (10 K) and liquid (18 K) p-H_2. The free-rotor transition line at 14.6 meV is observed only for the solid hydrogen. In solid-state, the hydrogen molecules are sufficiently isolated to behave as free rotors. For higher temperatures, the bulk phase melts and the INS spectrum of liquid H_2 is composed only of a broad maximum (recoil). (b) Free-rotor energy transitions scheme. The rotational transition J:1→0 correspond to 14.6 meV. A transition between the para (J=0) and ortho (J=1) spin states is observed due to the symmetry of the wavefunction.

5.1.1 The *Translational recoil of the dihydrogen* is observed in the INS spectra as a broad maximum centered close to 32 meV [20,22], which is a result of the transfer of momentum to the H_2 molecules without altering the non-translational degrees of freedom of the molecule, also observed in solid as well as for liquid dihydrogen (Fig. S10). It indicates the presence of a quantum liquid without well-defined adsorption positions.

5.1.2 The *Free-rotational quantum transition of H_2* is observed for solid-state due to the low intermolecular interactions and the small moment of inertia of the H_2 molecule, allowing the H_2 molecules to behave as isolated quantum rotors (free-rotor).

The rotational transition is given by the symmetry of the total wavefunction which implies that para-hydrogen (proton spins anti-parallel) can only exist in even *J* states while ortho-hydrogen (proton spins parallel) only exists for odd *J*. In the absence of significant external interactions, the rotational energy levels of hydrogen are given by

$$E = J(J+1)B_J \qquad \text{(Eq. 4)}$$

where *J* is the rotational quantum number and the rotational constant B_J = 7.35 meV.

The lowest energy transition occurs between J=1 and J=0, which is symmetry-allowed only by the spin interchange between ortho (J=1) and para (J=0) hydrogen and accounts for 14.6 meV [20,22,23]. This value coincides with the observed value for solid H_2 and indicates that the intermolecular interactions do not affect the free rotation [21,23]. Thus, the appearance of this signal in the adsorbed layer of H_2 is indeed not surprising.

Figure S10a (red) shows the INS spectrum of solid para-hydrogen at 10 K. The broad peak centered at 32 meV is related to the translational recoil of the hydrogen molecule. The peak centered at 14.6 meV is directly related to a free-rotational transition J:1→0. The rotational constant B_J=7.25 meV was experimentally obtained using the mean energy of rotational transition. Using the rotational constant B_J and the reduced mass of the hydrogen molecule, the length of the hydrogen molecule was calculated to be 0.756 Å.

5.2 Roto-vibrational transitions, hindered rotor (adsorbed hydrogen)

The INS spectra show three peaks centered at 10.6, 11.6, and 14.2 meV can be explained in terms of a hindered rotor. Figure S11 shows the energy transitions of the analysis assuming that the hydrogen molecules behave as hindered rotors with an apparent rotational constant B_J = 5.94. The energy transitions correspond to the INS results when the rotational barrier heights are V_ϕ = 0.4 meV and V_θ = 7.71 meV.

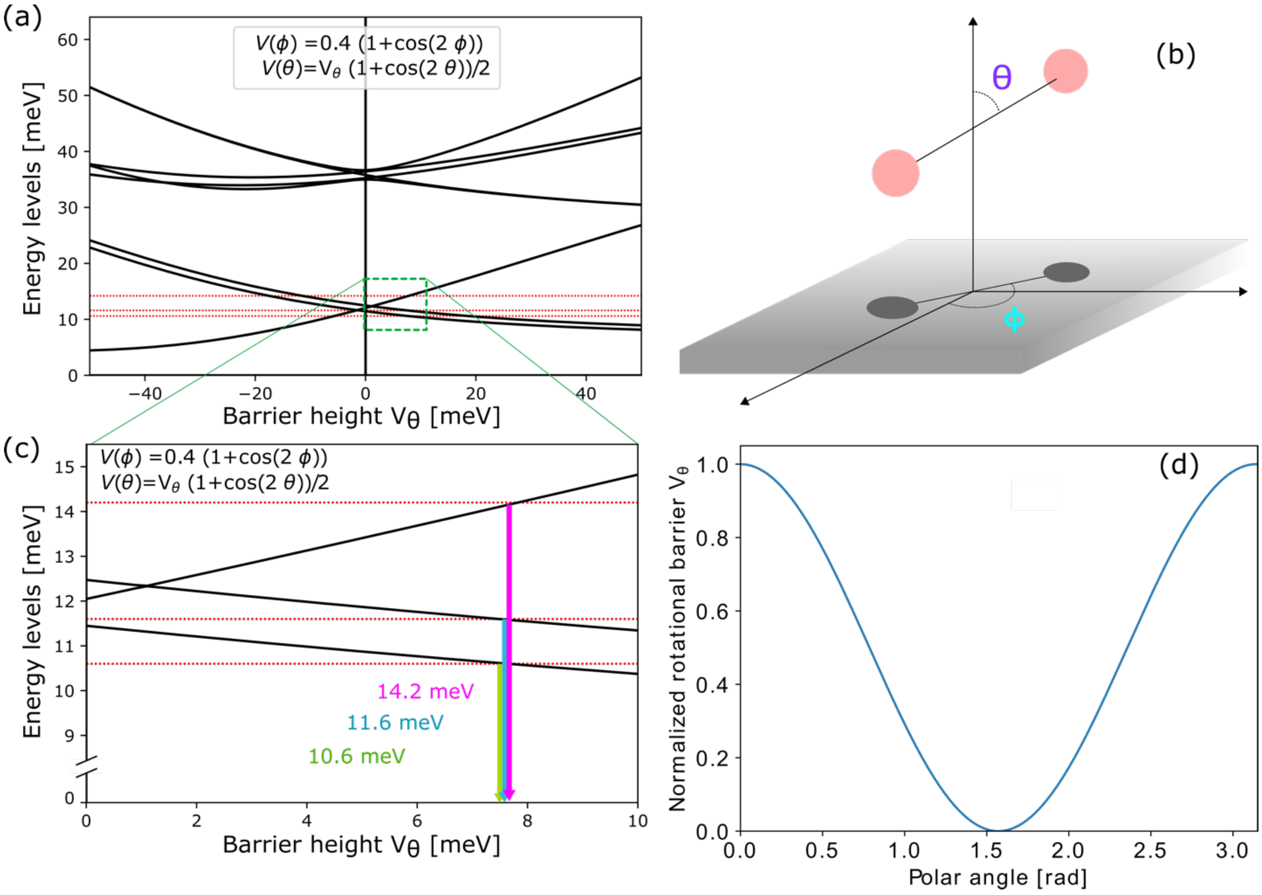

Figure S11. Hindered rotor levels of H_2 (a) Hindered rotor energy levels obtained as a function of the V_θ rotational barrier energy. Because of the symmetry of the rotational barrier, the free-rotor transition J:1→0 is split into three transitions depending on the barrier height. (b) The model for out-of-plane rotation of the hydrogen molecule near the silica surface. (c) Zoom of the hindered rotor energy levels, the experimental energies of the transitions are marked in red dotted lines. (d) Periodicity of the normalized rotational barrier as a function of the angle (θ). The energy transitions 10.59, 11.57 and 14.16 meV correspond to a rotational barrier V_ϕ = 0.4 meV and V_θ = 7.71 meV.

The details of the hindered rotation of adsorbed diatomic molecules can be found in MacRury's work [24] so we only summarize important equations in this section. The model is illustrated in Figure S11(b). The surface is seen as a continuous plane with adsorbed hydrogen. A hydrogen molecule sits on the surface at equilibrium distance Z. The hydrogen behaves like a hindered rigid rotor due to the quadrupole interaction between the dihydrogen molecule and the surface. The angle θ is defined as the angle between the normal surface passing through the center of mass of hydrogen and the molecular axis of the hydrogen.

The eigenvalues can be obtained by solving the Schrödinger equation,

$$\boldsymbol{\hat{H}\psi(\theta,\phi) = E\psi(\theta,\phi)} \qquad \textbf{(Eq. 5)}$$

The Hamiltonian is a combination of an unperturbed term and cross-terms.

$$\hat{H} = -(\hbar^2/2\mu)\nabla^2 + V(\theta,\phi) = H_{unperturbed} + H_{cross-terms} \qquad \text{(Eq. 6)}$$

where μ is the reduced mass of hydrogen, ∇ is the Laplacian operator, and $V(\theta,\phi)$ is the hindered potential (rotational barrier). The wavefunction $\psi(\theta,\phi)$ can be expressed by a product of $\Theta(\theta)$ and $\Phi(\phi)$

$$\psi(\theta,\phi) = \Theta(\theta)\Phi(\phi) = Y_{M,J}(\theta,\phi) \qquad \text{(Eq. 7)}$$

$$\Theta_{\mathrm{JM}}(\theta) = \sqrt{\frac{2J+1}{2}\frac{(J-M)!}{(J+M)!}}\,P_J^M(\cos(\theta)) \qquad \text{(Eq. 8)}$$

$$\Phi(\phi) = \frac{e^{iM\phi}}{\sqrt{2\pi}} \qquad \text{(Eq. 9)}$$

where $P_j^m(\cos(\theta))$ are the normalized associated Legendre polynomials.

The hindered potential can be written as

$$V(\theta,\phi) = V_\theta(1-cos(2\,\theta))/2 + V_\phi(1-cos(2\,\phi))/2 \qquad \text{(Eq. 10)}$$

Hence,

$$H_{J'M'|JM} = \left\langle \mathrm{Y}_{\mathrm{J'M'}}(\theta,\phi)\middle|\hat{\mathrm{H}}\middle|\mathrm{Y}_{\mathrm{JM}}(\theta,\phi)\right\rangle$$

$$= J(J'+1)B\,\delta_{JJ'} + \left\langle \mathrm{Y}_{\mathrm{J'M'}}(\theta,\phi)\middle|V(\theta,\phi)\middle|\mathrm{Y}_{\mathrm{JM}}(\theta,\phi)\right\rangle \qquad \text{(Eq. 11)}$$

where the unperturbed term (green) corresponds to the free-rotor, and cross-terms (red) can be separated in two angular terms, that can be solved independently.

$$\langle Y_{J'M'}(\theta,\phi)|V_\theta(\theta)V_\phi(\phi)|Y_{JM}(\theta,\phi)\rangle = \langle\Theta^*_{J'M'}(\theta)|V_\theta(\theta)|\Theta_{JM}(\theta)\rangle\ \langle\Phi^*_{M'}(\phi)|V_\phi(\phi)|\Phi_M(\phi)\rangle$$

$$= \int_0^\pi \Theta^*_{J'M'}(\theta)V_\theta(\theta)\Theta_{JM}(\theta)\sin\theta d\theta \cdot \int_0^{2\pi} \Phi^*_M(\phi)V_\phi(\phi)\Phi_M(\phi)d\phi \qquad \text{(Eq. 12)}$$

6. Theoretical analysis

6.1 Simulation of cross-sectional area

A molecule adsorbed on a flat surface that is in the x-y-plane (2D structure) surrounded by other molecules of the same kind can be described by the model Hamiltonian:

$$\hat{H} = -\frac{\hbar^2}{2m}\Delta + \underbrace{\sum \hat{V}_M(\vec{r}-\vec{r_i}) + \hat{V}_s}_{:=\hat{V}_{tot}(\vec{r})} \qquad \text{(Eq. 13)}$$

with

$$\hat{V}_M(\vec{r}) = D_e\left(\left(e^{-\alpha\left(\sqrt{x^2+y^2}-R_e\right)}-1\right)^2-1\right) \qquad \text{(Eq. 14)}$$

where $\hat{V}_M(\vec{r})$ is the spherically symmetric Morse-potential that describes the intermolecular interaction, $\vec{r_i}$ denotes the positions of the surrounding molecular centers and V_s the average adsorption potential of the surface. D_e, α, and R_e are fitted by the *ab initio* results at the CCSD(T)/aug-cc-pVQZ level. The fitting procedure can be found in Section 5.2. The total potential felt by the central H_2 molecule is simply the sum of the pair-potentials and the adsorption potential (see Figures S12 and S13 for a 1D sketch of the confining potential). As it is of interest how much the central H_2 can be compressed by the surrounding molecules before becoming unstable, we assume a hexagonal 2D network (hxl), where all next neighbor distances are equal and treated as parameter a.

In a real space representation, the stationary Schrödinger equation can be written as:

$$\left(-\frac{\hbar^2}{2m}\Delta + \hat{V}_{tot}(\vec{r})\right)\varphi(\vec{r}) = E\varphi(\vec{r}) \qquad \text{(Eq. 15)}$$

substituting $\lambda^2(\vec{r}) = \frac{2m\hat{V}_{tot}}{\hbar^2}$ and $\varepsilon = \frac{2mE}{\hbar^2}$ leads to:

$$\left(\frac{\partial^2}{\partial x^2} + \frac{\partial^2}{\partial y^2} - \lambda^2(x,y) + \varepsilon\right)\varphi(x,y) = 0 \qquad \text{(Eq. 16)}$$

The numerical solution of this partial differential equation (PDE) is obtained using the Octave software[25], employing a 5-point stencil central differences scheme[26-28]. The discretization of the Laplacian is straight-forward:

$$f'(x) = \lim_{h\to 0}\frac{f(x+h) - f(x)}{h} = \lim_{h\to 0}\frac{f(x) - f(x-h)}{h} = \lim_{h\to 0}\frac{f(x+h) - f(x-h)}{2h} \qquad \text{(Eq. 17)}$$

$$f''(x) = \frac{d}{dx}\lim_{h\to 0}\frac{f(x+h) - f(x)}{h} = \frac{d}{dx}\lim_{h\to 0}\frac{f(x) - f(x-h)}{h} = \lim_{h\to 0}\frac{f(x+h) + f(x-h) - 2f(x)}{h^2} \qquad \text{(Eq. 18)}$$

Considering that the infinitesimal displacements h are not necessarily equal in different directions (boundaries), we use

$$h \in [\Delta x_+, \Delta x_-, \Delta y_+, \Delta y_-] \qquad \text{(Eq. 19)}$$

which leads to:

$$\begin{aligned} 0 = {} & \frac{\varphi(x+\Delta x_+, y) - \varphi(x,y)}{\Delta x_+^2} + \frac{\varphi(x-\Delta x_-, y) - \varphi(x,y)}{\Delta x_-^2} \\ & + \frac{\varphi(x, y+\Delta y_+) - \varphi(x,y)}{\Delta y_+^2} + \frac{\varphi(x, y-\Delta y_-) - \varphi(x,y)}{\Delta y_-^2} \\ & -(\lambda^2 - \varepsilon)\varphi(x,y) \end{aligned} \qquad \text{(Eq. 20)}$$

using the abbreviation $\varphi_{x/y}^{\pm} := \varphi\,(x \pm \Delta x_{\pm}, y) / \varphi\,(x, y \pm \Delta y_{\pm})$ and re-grouping the expression gives:

$$\underbrace{\left(\lambda^2 - \varepsilon + \frac{1}{\Delta x_+^2} + \frac{1}{\Delta x_-^2} + \frac{1}{\Delta y_+^2} + \frac{1}{\Delta y_-^2}\right)}_{:=LHS-\epsilon} \varphi(x,y) = \underbrace{\left(\frac{\varphi_x^+}{\Delta x_+^2} + \frac{\varphi_x^-}{\Delta x_-^2} + \frac{\varphi_y^+}{\Delta y_+^2} + \frac{\varphi_y^-}{\Delta y_-^2}\right)}_{:=RHS} \quad \text{(Eq. 21)}$$

i. e.:

$$\varphi(x,y) = \frac{RHS}{LHS-\varepsilon} \qquad \text{and} \qquad \varepsilon = LHS - \frac{RHS}{\varphi(x,y)}$$

As an initial guess for the wavefunction φ, a 2D Gaussian distribution has been used. In each relaxation step, a new wavefunction φ and a new energy ε are obtained. For the correct wavefunction φ that solves the PDE, the energy ε is the same on every grid-point. Vice versa, test functions that are not the correct solution to the PDE lead to different values of ε on each grid-point at the i-th iteration: $\varepsilon_{x,y}^i$. The mean value for the energy is therefore simply given by:

$$\varepsilon^i = \frac{1}{N} \sum \varepsilon_{x,y}^i \quad \text{(Eq. 22)}$$

The root mean square deviation (RMSD) is given by:

$$\Delta\varepsilon_{RMSD}^i = \sqrt{\frac{1}{N} \sum \left(\varepsilon^i - \varepsilon_{x,y}^i\right)^2} \quad \text{(Eq. 23)}$$

The iteration is stopped when the change in energy between successive iteration steps ($E^{i+1} - E^i$) and the RMSD of the energy drop below 10^{-3} meV. For the calculation, periodic boundary conditions have been considered to avoid divisions by very small numbers (although the probability density of the central H_2 molecule is located in the center of the hexagon and quickly tends to 0 when going to the edge of the hexagon), and for the calculation of the energy only $\varphi(x,y)$ with $|\varphi(x,y)| \geq 10^{-6} \cdot max\big(\varphi(x,y)\big)$.

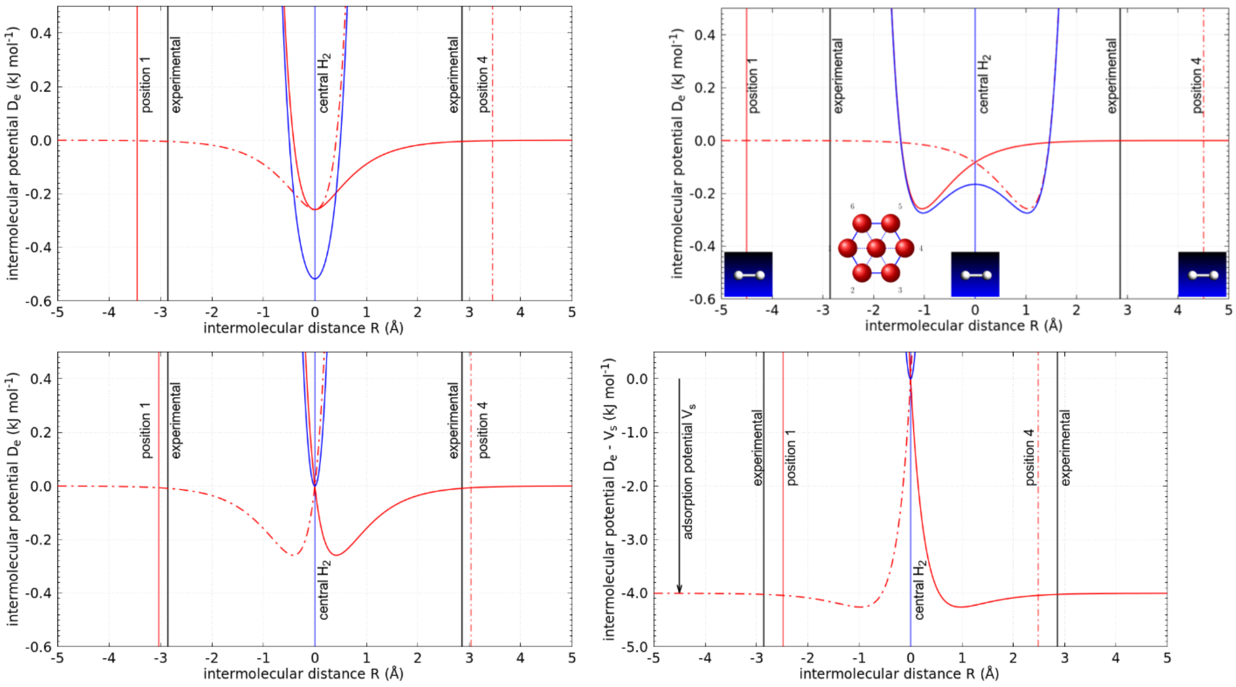

Figure S12. 1D sketch of the effect of confining a molecule between two other molecules. The red curves show the Morse-potential between the central H_2 molecule and one of the confining H_2 molecules, the blue curve shows the total potential that is felt by the central H_2 molecule, the vertical red lines indicate the positions of the surrounding H_2 molecules, and the vertical black line shows the experimentally measured value. Top left panel: distance between neighbors is bigger than the equilibrium distance (of liquid H_2). Top right panel: distance between neighbors is the equilibrium distance of the H_2 dimer. Bottom left panel: maximum compression without additional potential. Bottom right panel: maximum compression with additional 4 kJ/mol adsorption potential.

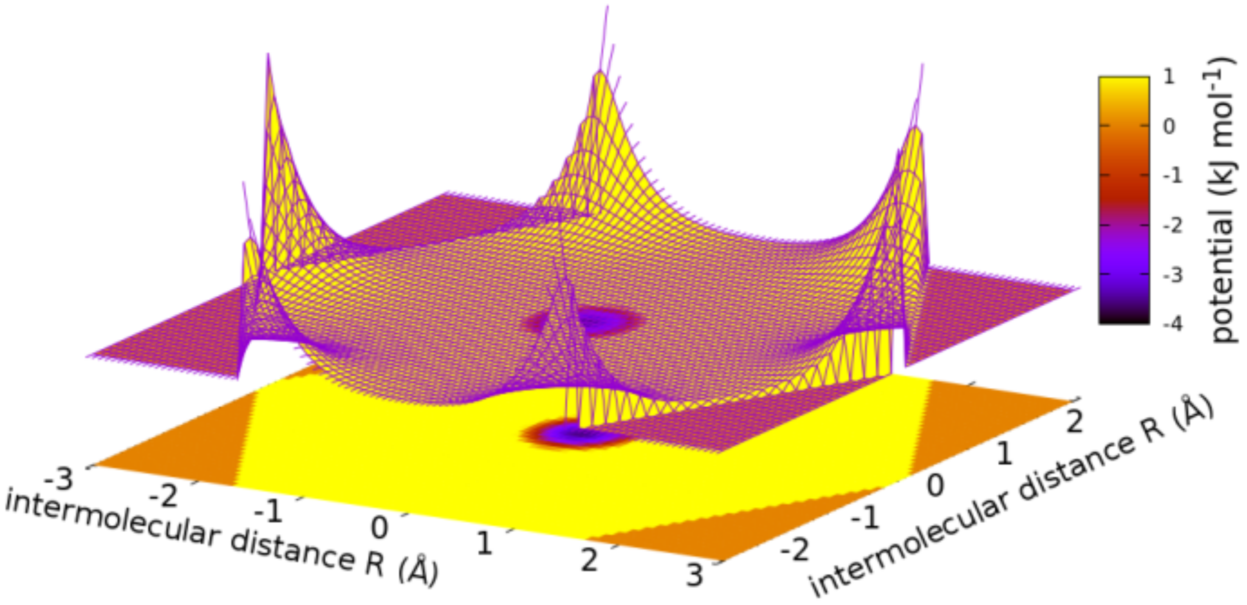

Figure S13. **Potential of the central H_2 molecule in a hexagonal cell.** For intermolecular distances equal or below the equilibrium distance between H_2 molecules in a liquid state, the potential is only binding in a small region in the center of the hexagon (some 10 pm).

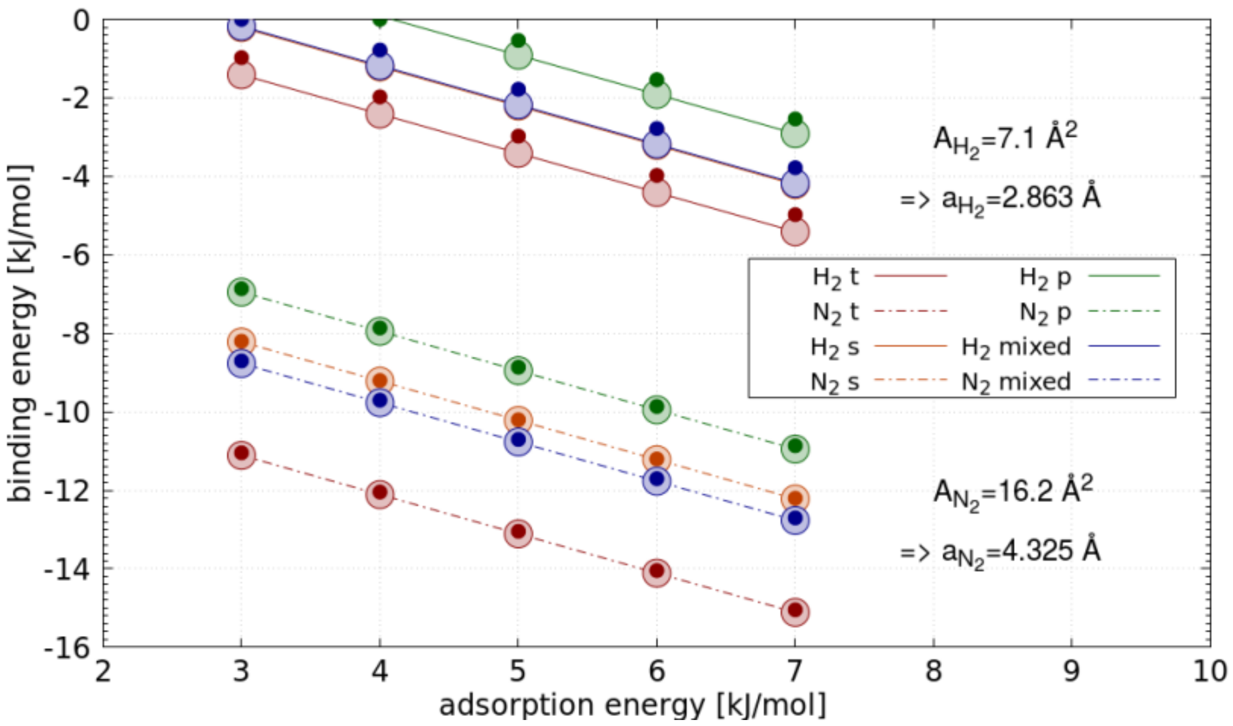

Figure S14. Calculated energy binding as a function of the adsorption potential. The binding energy for a fixed area per molecule of 7.1 Å^{-2} (the experimental area) depends on the adsorption potential and the relative orientation of the next neighbors. The lines and empty circles correspond to a classic consideration, while the small filled dots include nuclear quantum effects. In the range between 3-4 kJ/mol adsorption potential, For the most stable and the mixed structure the binding energy is between 1 and 2 kJ/mol.

6.2 Fitting Morse potential parameters.

We here describe the details of the fitting process. To model the interaction energy between two hydrogen molecules Morse potential was used,

$$V^X = D_e^X\left(e^{-\alpha^X(R-R_e^X)} - e^{-\alpha^X(R-R_e^X)}\right) \qquad \text{(Eq. 24)}$$

The D_e^X is the well-depth, R_e^X is the equilibrium distance between the center of mass of hydrogen molecules, and α^X controls the well-width of the potential. In close packing, orientation, denoted as X, has to be taken carefully since it is a significant factor of potential. We consider three possible configurations, i.e. p, T, and s. They are illustrated in Figure S15.

The first step is to calculate the energies of H_2 dimers along different separations. (circles in Figure S15) The first-principles calculations were performed at CCSD(T)/aug-cc-pVQZ level with the quantum chemistry package ORCA[29]. Parameters of Morse potential were then fitted with those data.

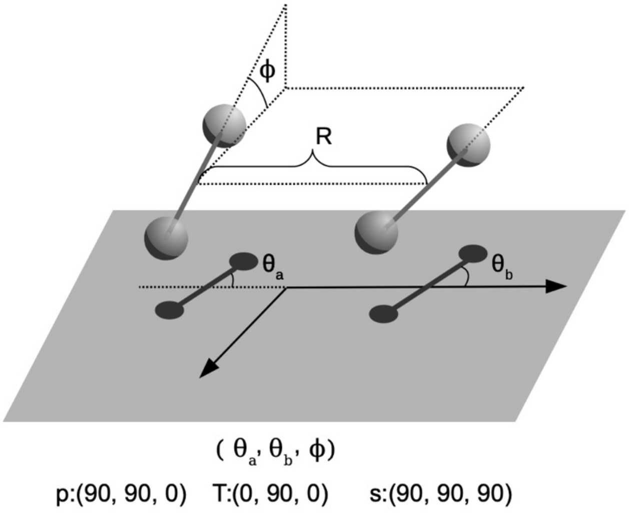

Figure S15. Orientation configuration of a diatomic molecule. We used three different configurations for diatomic molecules and the corresponding angular coordinates (θ_a, θ_b, ϕ_a=ϕ_b).

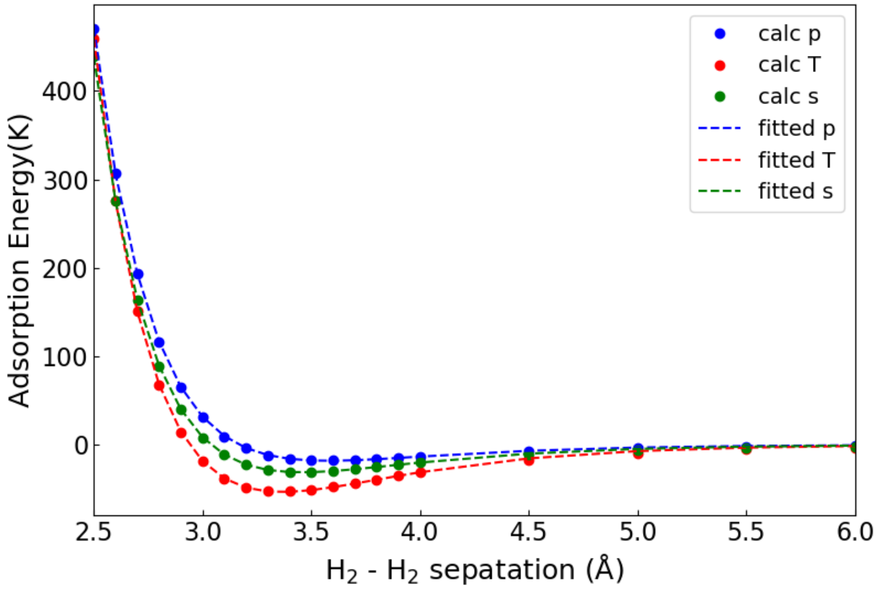

Figure S16. Morse potential curve for three configurations. 'Calc' denotes the calculation results at CCSD(T)/aug-cc-pVQZ level while fitted represents the fitting results.

7. MD and Path Integral Molecular Dynamics simulations

A silica surface ($Si_{72}O_{144}$) layer was geometrically optimized. Our model considers a cubic cell of 24 x 24 Å. Figure S17 shows several views of the silica layer.

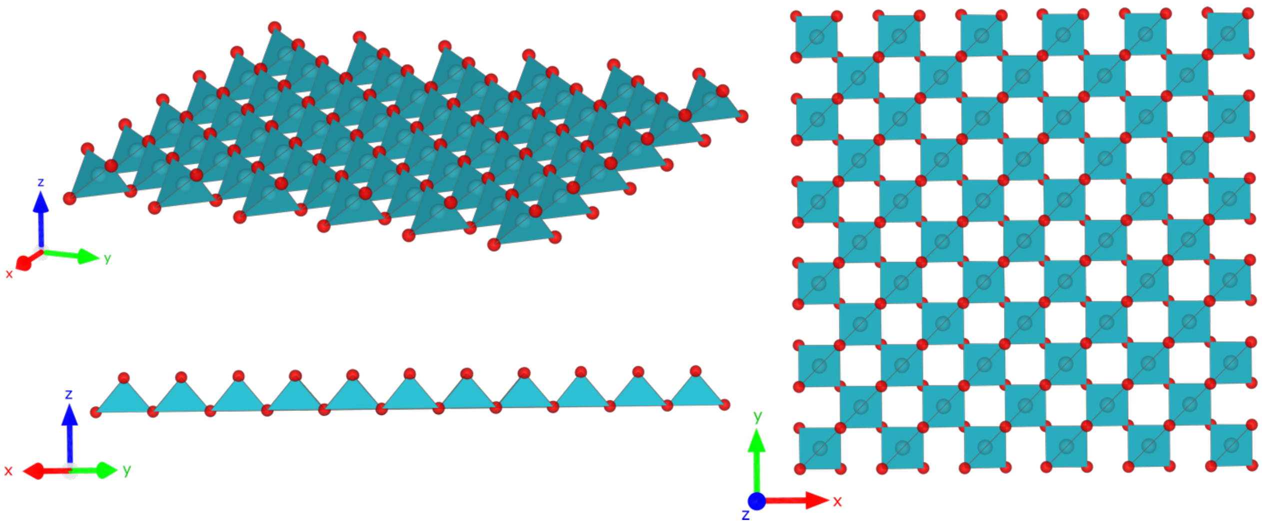

Figure S17. Silica layer representation. A silica surface ($Si_{72}O_{144}$) layer in a cubic cell (24 x 24 Å) was used geometrically optimized. We used the same surface for all our simulations.

7.1 Argon adsorption simulation

Molecular dynamics (MD) simulation was made for a layer of argon at the temperature of 90 K and each step in the simulation corresponds to 2 fs. The simulation assumes the silica and oxygen atoms are completely fixed while the argon atoms are free to move. The final step of the simulation (27540 fs) is shown in Fig. S18b. The adsorbed layer remains composed of 42 argon atoms that are desorbed and adsorbed during the simulation (Fig. S18c). The pair-distribution function was obtained using the positions of the last 500 time-steps of the simulation, yielding an inter-atomic distance of 3.92 Å (Fig. S18e), which is reasonably close to the bulk-liquid argon (4.07 Å), calculated using Eq. 2, 3 and the bulk-liquid density of argon (1395 kg m^{-3})

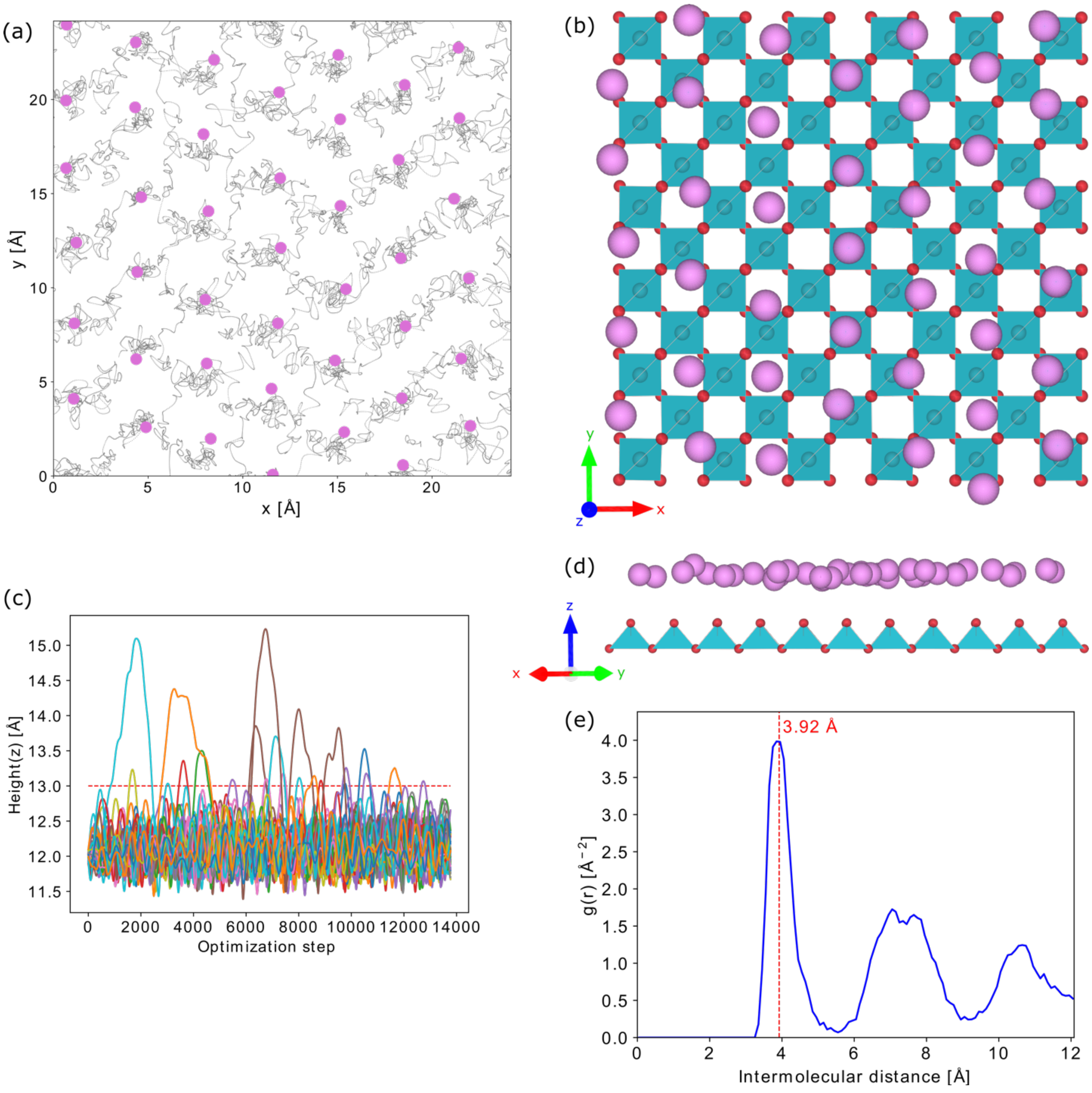

Figure S18. Molecular dynamics (MD) of adsorbed Argon on silica. The simulation was run for 14000 steps assuming a temperature of 90 K. (a) XY-plane view of the argon atom's path of motion, the position of the atoms in the last step is marked in purple. (b) XY-plane view of the final frame of the simulation, the Ar atoms form a hexagonal arrangement. (c) z-axis position of all molecules as a function of the optimization step, representing the height of the Ar atoms to the silica layer. (d) Lateral view of the final frame of the simulation. (e) Histogram of the distances between the Ar atoms, the first neighbors' distance is 3.92 Å.

7.2 Hydrogen adsorption simulation

Path Integral Molecular Dynamics (PIMD) was used to simulate the hydrogen adsorbed layer at boiling temperatures. The temperature for the simulation was 30 K, and the time step was 1 fs.

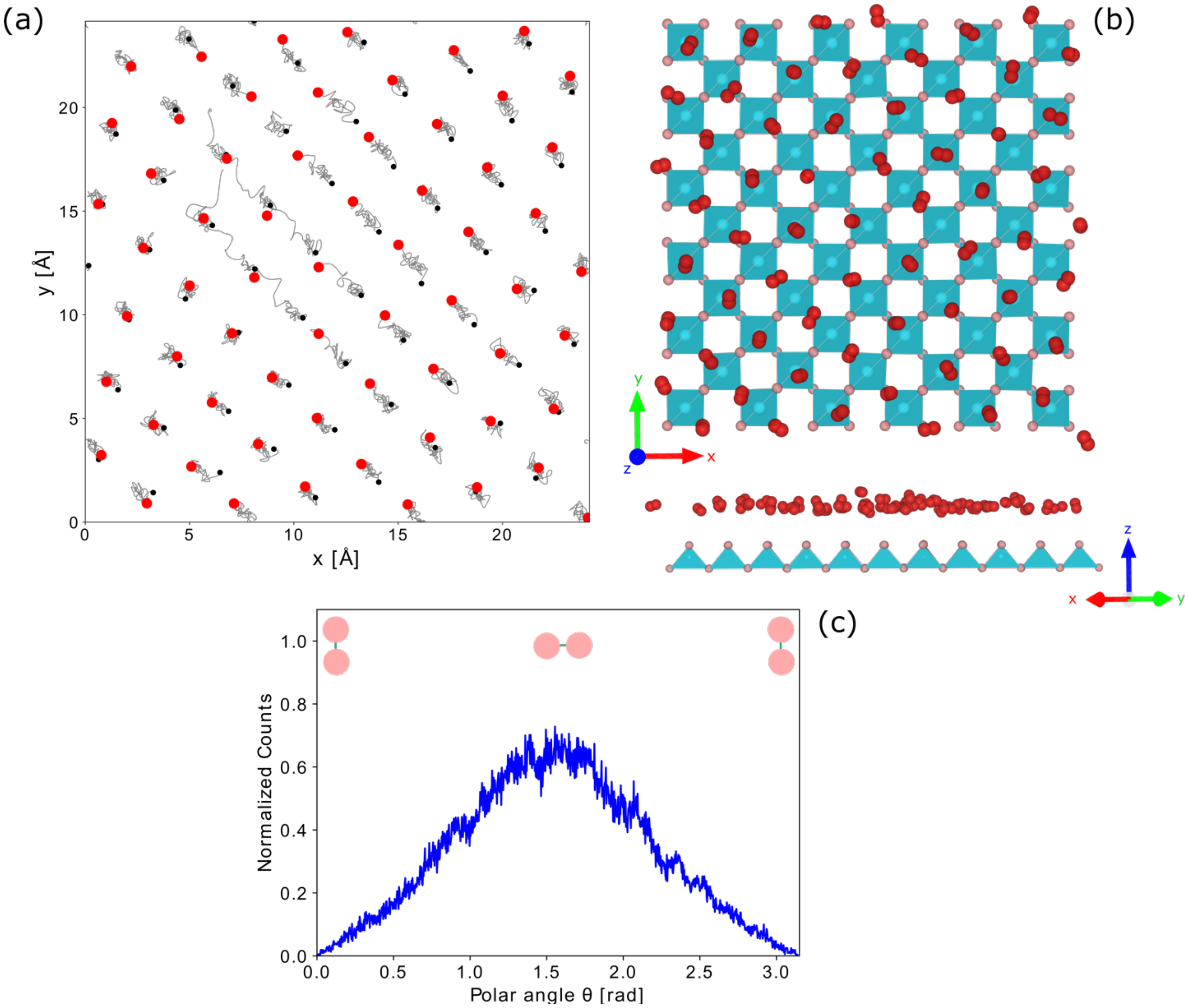

Figure S19. Path Integral Molecular Dynamics (PIMD) of hydrogen adsorbed on silica The simulation was run 2500 steps at a temperature of 30 K. (a) XY-plane view of the H_2 molecule's path of motion, the position of the atoms in the first step is marked in black and last step is marked in red. The initial configuration of the H_2 molecules on a silica surface corresponds to a molecular dynamics (MD) equilibrated configuration. The final positions (red dots) of the H_2 molecules show a hexagonal pattern. (b) Vertical and lateral view of the final configuration of the simulation. The lateral view shows that all molecules in the final steps are in the first adsorbed layer (monolayer). (c) Orientation distribution of the H_2 molecules as a function of the polar angle. The H_2 molecules are preferentially oriented parallel to the surface.

The MD equilibrated configuration was used as the starting point, and 2500 steps were simulated. The final step of the simulation is shown in Fig. S19b. The adsorbed layer remains composed of 63 dihydrogen molecules that are desorbed and adsorbed during the simulation (Fig. 4b). The pair-distribution function was obtained using the positions of all steps of the simulation, yielding an inter-atomic distance of 3.2 Å (Fig. 4c), which is reasonably close to the experimental findings (2.9 Å) (see Section 2.4). The orientation of the molecules was calculated for all steps of the simulation. Figure S19c shows the histogram of the polar angle θ. The molecules have a predominant orientation parallel to the surface $\theta = {}^{\pi}/_{2}$.

8. Data availability

The source data for figures and supplementary material can be found at the Zenodo repository (doi: 10.5281/zenodo.6623388) [30].

9. Supplementary References

1 Schaeffer, W. D., Smith, W. R. & Wendell, C. B. The Adsorption of Helium on Carbon Black at Liquid Helium Temperatures. *J. Am. Chem. Soc.* **71**, 863-867, doi:10.1021/ja01171a027 (1949).

2 Pace, E. L. & Siebert, A. R. Heat of Adsorption of Parahydrogen and Orthodeuterium on Graphon. *J. Phys. Chem.* **63**, 1398-1400, doi:10.1021/j150579a014 (1959).

3 Brewer, D. F. & Champeney, D. C. Sorption of Helium and Nitrogen on Vycor Porous Glass. *Proceedings of the Physical Society* **79**, 855 (1962).

4 Huber, T. E. & Huber, C. A. Vibrational spectroscopy of H2 in porous Vycor glass: First evidence for the bilayer structure. *Phys. Rev. Lett.* **59**, 1120-1123, doi:10.1103/PhysRevLett.59.1120 (1987).

5 Huber, T. E. & Huber, C. A. Vibrational spectroscopy of porous Vycor glass: surface hydroxyl perturbations upon adsorption of hydrogen. *J. Phys. Chem.* **94**, 2505-2511, doi:10.1021/j100369a053 (1990).

6 Setoyama, N. & Kaneko, K. Density of He adsorbed in micropores at 4.2K. *Adsorption* **1**, 165-173, doi:10.1007/bf00705003 (1995).

7 J. Edler, K., A. Reynolds, P., J. Branton, P., R. Trouw, F. & W. White, J. Structure and dynamics of hydrogen sorption in mesoporous MCM-41. *Journal of the Chemical Society, Faraday Transactions* **93**, 1667-1674, doi:10.1039/A607878H (1997).

8 Tanaka, H. *et al.* Quantum Effects on Hydrogen Adsorption in Internal Nanospaces of Single-Wall Carbon Nanohorns. *J. Phys. Chem. B* **108**, 17457-17465, doi:10.1021/jp048603a (2004).

9 Prisk, T. R., Bryan, M. S. & Sokol, P. E. Diffusive and rotational dynamics of condensed n-H2 confined in MCM-41. *Phys. Chem. Chem. Phys.* **16**, 17960-17974, doi:10.1039/C4CP02281E (2014).

10 Huber, T. E. & Huber, C. A. Adsorption of hydrogen on porous Vycor glass. *J. Low Temp. Phys.* **80**, 315-323, doi:10.1007/bf00683637 (1990).

11 Hussain, M., Fino, D. & Russo, N. Development of modified KIT-6 and SBA-15-spherical supported Rh catalysts for N2O abatement: From powder to monolith supported catalysts. *Chem. Eng. J.* **238**, 198-205, doi:10.1016/j.cej.2013.06.032 (2014).

12 Porcheron, F., Thommes, M., Ahmad, R. & Monson, P. A. Mercury Porosimetry in Mesoporous Glasses: A Comparison of Experiments with Results from a Molecular Model. *Langmuir* **23**, 3372-3380, doi:10.1021/la063080e (2007).

13 Gille, W., Enke, D. & Janowski, F. in *Stud. Surf. Sci. Catal.* Vol. 144 (eds F. Rodriguez-Reinoso, B. McEnaney, J. Rouquerol, & K. Unger) 593-600 (Elsevier, 2002).

14 Thommes, M. *et al.* Physisorption of gases, with special reference to the evaluation of surface area and pore size distribution (IUPAC Technical Report). *Pure Appl. Chem.* **87**, 1051-1069, doi:10.1515/pac-2014-1117 (2015).

15 Gurvich, L. Physico-chemical attractive force. *J. Phys. Chem. Soc. Russ.* **47**, 805-827 (1915).

16 Thommes, M. & Cychosz, K. A. Physical adsorption characterization of nanoporous materials: progress and challenges. *Adsorption* **20**, 233-250, doi:10.1007/s10450-014-9606-z (2014).

17 Monson, P. A. Understanding adsorption/desorption hysteresis for fluids in mesoporous materials using simple molecular models and classical density functional theory. *Microporous Mesoporous Mater.* **160**, 47-66, doi:https://doi.org/10.1016/j.micromeso.2012.04.043 (2012).

18 Rouquerol, F., Rouquerol, J. & Sing, K. S. W. *Adsorption by powders and porous solids: principles, methodology, and applications*. (Academic Press, 1999).

19 Lowell, S., Shields, J. E., Thomas, M. A. & Thommes, M. in *Characterization of Porous Solids and Powders: Surface Area, Pore Size and Density* Vol. 16 *Particle Technology Series* 58-81 (Springer Netherlands, 2004).

20 Mitchell, P. C. H., Parker, S. F., Ramirez-Cuesta, A. J. & Tomkinson, J. *Vibrational spectroscopy with neutrons : with applications in chemistry, biology, materials science and catalysis*. (World Scientific, 2005).

21 Silvera, I. F. The solid molecular hydrogens in the condensed phase: Fundamentals and static properties. *Rev. Mod. Phys.* **52**, 393-452, doi:10.1103/RevModPhys.52.393 (1980).

22 Georgiev, P. A. *et al.* Hydrogen site occupancies in single-walled carbon nanotubes studied by inelastic neutron scattering. *J Phys-Condens Mat* **16**, L73-L78, doi:10.1088/0953-8984/16/8/l01 (2004).

23 Requena, A., Peña, R. & Serna, A. Perturbation for a rigid rotator in an electric field. *Int. J. Quantum Chem.* **22**, 1263-1270, doi:10.1002/qua.560220609 (1982).

24 Macrury, T. B. & Sams, J. R. Hindered Rotation of Adsorbed Diatomic Molecules .1. Eigenvalues and Eigenstates of Hindered Rotator. *Mol. Phys.* **19**, 337-&, doi:10.1080/00268977000101351 (1970).

25 Eaton, J. W., Bateman, D., Hauberg, S. & Wehbring, R. GNUOctave Version 4.0.0 Manual: A High-Level Interactive Language for Numerical Com-putations. *Fourth. CreateSpace Independent Publishing Platform.*, https://octave.org/doc/interpreter (2015).

26 William, H. P. Numerical recipes. *The art of scientific computing* **745** (2007).

27 Lang, C. B. & Pucker, N. *Mathematische Methoden in der Physik*. (Springer Berlin Heidelberg, 2016).

28 Sauer, T. *Numerical Analysis*. Vol. 2nd edition (Pearson, 2012).

29 Neese, F. Software update: the ORCA program system, version 4.0. *Wiley Interdisciplinary Reviews: Computational Molecular Science* **8**, e1327, doi:10.1002/wcms.1327 (2018).

30 Balderas-Xicohténcatl, R. *et al.* Formation of super-dense hydrogen monolayer on mesoporous silica (v1.0) [Data set]. *Zenodo*, doi:10.5281/zenodo.6623388 (2022).
